# PET imaging of the serotonin 1A receptor in major depressive disorder: Hierarchical multivariate analysis of [ ^11^ C]WAY100635 overcomes outcome measure discrepancies

**DOI:** 10.1162/imag_a_00328

**Published:** 2024-10-25

**Authors:** Granville J. Matheson, Francesca Zanderigo, Jeffrey M. Miller, Elizabeth A. Bartlett, J. John Mann, R. Todd Ogden

**Affiliations:** Department of Psychiatry, Columbia University, New York, NY, United States; Department of Biostatistics, Columbia University Mailman School of Public Health, New York, NY, United States; Molecular Imaging and Neuropathology Area, New York State Psychiatric Institute, New York, NY, United States; Department of Clinical Neuroscience, Center for Psychiatry Research, Karolinska Institutet and Stockholm County Council, Stockholm, Sweden

**Keywords:** positron emission tomography, serotonin 1A receptor, major depressive disorder, pharmacokinetic modelling

## Abstract

The serotonin 1A receptor has been linked to both the pathophysiology of major depressive disorder (MDD) and the antidepressant action of serotonin reuptake inhibitors. Most PET studies of the serotonin 1A receptor in MDD used the receptor antagonist radioligand, [carbonyl-C11]WAY100635; however, the interpretation of the combined results has been contentious owing to reports of higher or lower binding in MDD with different outcome measures. The reasons for these divergent results originate from several sources, including properties of the radiotracer itself, which complicate its quantification and interpretation; as well as from previously reported differences between MDD and healthy volunteers in both reference tissue binding and plasma-free fraction, which are typically assumed not to differ. Recently, we have developed two novel hierarchical multivariate methods which we validated for the quantification and analysis of [C11]WAY100635, which show better accuracy and inferential efficiency compared to standard analysis approaches. Importantly, these new methods should theoretically be more resilient to many of the factors thought to have caused the discrepancies observed in previous studies. We sought to apply these methods in the largest [C11]WAY100635 sample to date, consisting of 160 individuals, including 103 MDD patients, of whom 50 were not-recently-medicated and 53 were antidepressant-exposed, as well as 57 healthy volunteers. While the outcome measure discrepancies were substantial using conventional univariate analysis, our multivariate analysis techniques instead yielded highly consistent results across PET outcome measures and across pharmacokinetic models, with all approaches showing higher serotonin 1A autoreceptor binding potential in the raphe nuclei of not-recently-medicated MDD patients relative to both healthy volunteers and antidepressant-exposed MDD patients. Moreover, with the additional precision of estimates afforded by this approach, we can show that while binding is also higher in projection areas in this group, these group differences are approximately half of those in the raphe nuclei, which are statistically distinguishable from one another. These results are consistent with the biological role of the serotonin 1A autoreceptor in the raphe nuclei in regulating serotonin neuron firing and release, and with preclinical and clinical evidence of deficient serotonin activity in MDD due to over-expression of autoreceptors resulting from genetic and/or epigenetic effects. These results are also consistent with downregulation of autoreceptors as a mechanism of action of selective serotonin reuptake inhibitors. In summary, the results using multivariate analysis approaches, therefore, demonstrate both face and convergent validity, and may serve to provide a resolution and consensus interpretation for the disparate results of previous studies examining the serotonin 1A receptor in MDD.

## 1 Introduction

The serotonin (5-HT) 1A receptor (5-HT_1A_R) serves as a postsynaptic heteroreceptor in most of the brain, and as a presynaptic autoreceptor in the raphe nuclei where serotonin neuron cell bodies are located (Barnes & Sharp, 1999;Hoyer et al., 2002). Owing to its autoreceptor function, it is able to centrally regulate firing rates of serotonin neuron and the release levels of 5-HT throughout the brain. The 5-HT_1A_R has been implicated in the pathophysiology of several psychiatric and neurological disorders (Akimova et al., 2009;Arango et al., 2001;Kepe et al., 2006;Savic et al., 2004;Tiihonen et al., 2004), and of major depressive disorder (MDD) in particular (Boldrini et al., 2008;Kaufman et al., 2016). The autoreceptor is implicated in both the pathophysiology of MDD, as well as the antidepressant action of many antidepressant medications (Blier & De Montigny, 1983;Gray et al., 2013;Richardson-Jones et al., 2010). For these reasons, the 5-HT_1A_R has been and remains of substantial research interest and importance for human molecular imaging studies of MDD patients. In humans,*in vivo*quantification of 5-HT_1A_R is performed using PET, with most studies using [carbonyl-C11]WAY100635, hereon referred to as [C11]WAY100635, due to its high affinity, target selectivity and favorable kinetics (Farde et al., 1997;Kumar & Mann, 2007;Osman et al., 1998). Over the last two decades, numerous PET studies have examined [C11]WAY100635 binding comparing MDD patients and healthy volunteers (HV)—however, results have been mixed, making their interpretation contentious (Parsey et al., 2010;Shrestha et al., 2012). The major source of this disagreement regarding the direction of the findings concerns the quantification of [C11]WAY100635 binding and the use of different outcome measures across different studies (for more details, seeSupplementary Materials S1).

The study of the 5-HT_1A_R using PET in humans has been particularly challenging due to both the properties of the [C11]WAY100635 radioligand and the neurobiology of the 5-HT_1A_R. The majority of studies have used reference tissue (i.e., indirect) approaches to estimate$BP_{\text{ND}}$using the cerebellum as a reference region, without collecting arterial plasma. However, the interpretation of these studies is complicated by the fact that the extremely low non-displaceable binding of [C11]WAY100635 renders the cerebellum disproportionately sensitive to the small degree of contamination from radiometabolites (Osman et al., 1998;Shrestha et al., 2012). Because the relationship of$BP_{\text{ND}}$with non-displaceable binding is proportional rather than subtractive (as it is for$BP_{\text{P}}$or$BP_{\text{F}}$), indirect estimation of$BP_{\text{ND}}$is particularly prone to bias in cerebellar binding estimates, and this contamination is thought to produce inaccurate estimates. Moreover, studies have shown not only that there is a non-negligible degree of specific binding of [C11]WAY100635 in the cerebellar grey matter which differs between individuals (Hirvonen et al., 2007;Parsey et al., 2005,^2010^), but also that cerebellar binding is higher in MDD patients than healthy volunteers (HV) (Parsey et al., 2010). Together with the low non-displaceable binding of [C11]WAY100635 and the high sensitivity of$BP_{\text{ND}}$to small biases in cerebellar binding, group differences in cerebellar grey matter binding induce artificially lower estimates of$BP_{\text{ND}}$in MDD patients when it is used as a reference region (Parsey et al., 2010). Cerebellar white matter has, therefore, been recommended as the preferred reference region (Hirvonen et al., 2007;Parsey et al., 2005); however, this region is even more affected by radiometabolites due to its lower binding. On the basis of these issues, caution has been urged generally for the interpretation of$BP_{\text{ND}}$for the analysis of [C11]WAY100635 data (Hirvonen et al., 2007;Parsey et al., 2005,^2010^;Shrestha et al., 2012).

Indirect quantification of$BP_{\text{P}}$and$BP_{\text{F}}$using arterial plasma allows researchers to minimise the influence of these biases owing to the subtractive, as opposed to proportional, relationship of these outcomes with cerebellar binding. In the subset of studies in which arterial plasma samples were collected, results using$BP_{\text{P}}$have been mixed (Hirvonen et al., 2008;Meltzer et al., 2004;Parsey et al., 2006), while results using$BP_{\text{F}}$have been more consistent.Parsey et al. (2006)observed globally higher [C11]WAY100635$BP_{\text{F}}$in unmedicated MDD patients compared to HV, which was later replicated in two subsequent studies (Miller et al., 2013;Parsey et al., 2010). However, interpretation of these studies is not straightforward either: although differences were observed using$BP_{\text{F}}$,$f_{\text{P}}$differed significantly between MDD and control groups (Parsey et al., 2010). Reasons for group differences in$f_{\text{P}}$are not well understood, and it cannot be known whether these differences could have been caused by some experimental confound such as experimental drift, or whether they were biological. Unfortunately however,$BP_{\text{F}}$can only be estimated when$f_{\text{P}}$is measured, and only our group has done so in human PET studies with [C11]WAY100635. There are therefore no other datasets in which to probe these$f_{\text{P}}$effects. Setting$f_{\text{P}}$aside, group differences were observed using$BP_{\text{P}}$inParsey et al. (2006), but these were not replicated inParsey et al. (2010), whileHirvonen et al. (2008)andMeltzer et al. (2004)found differences in the opposite direction. On the other hand, no differences were found using$BP_{\text{ND}}$using the cerebellar white matter as a reference region. The aforementioned issues are restricted to*in vivo*molecular imaging; however,*post mortem*autoradiography studies of the 5-HT_1A_in MDD have also yielded mixed results, thought to be due to confounding from medication effects, comorbidities, and postmortem interval among others (Kaufman et al., 2016). Confounding from medication effects may also be important for interpretation of the*in vivo*studies too, although most studies with arterial plasma sampling focused on unmedicated or not-recently-medicated patients (Hirvonen et al., 2008;Miller et al., 2013;Parsey et al., 2006,^2010^). In summary, this research question remains unresolved, with some review articles concluding that 5-HT_1A_R exhibits higher binding in unmedicated MDD patients relative to HV (Kaufman et al., 2016;Shrestha et al., 2012), while others conclude that binding is lower in MDD (Savitz & Drevets, 2013;Wang et al., 2016)—although the latter makes use of meta-analysis of study outcomes across different outcome measures (including indirect$BP_{\text{ND}}$), reference regions, and even diagnoses, which we would assert to be an inappropriate application of meta-analysis for this research question in particular. Besides the contrasting clinical conclusions arrived at by different surveys of the literature, the methodological questions surrounding group differences in reference quantities, and how best to contend with them have remained unclear (Parsey et al., 2010;Shrestha et al., 2012).

We have recently developed two new methods for the analysis of PET data which make use of a hierarchical multivariate modelling approach: SiMBA (Simultaneous Multifactor Bayesian Analysis) for combined quantification and statistical analysis (Matheson & Ogden, 2022), and a simplified version of this approach called PuMBA (Parameters undergoing Multivariate Bayesian Analysis) for multivariate statistical analysis following prior quantification (Matheson & Ogden, 2023). By using a hierarchical modelling strategy, SiMBA improves the accuracy and identifiability of estimated parameters, and is therefore able to reliably quantify binding potential directly from rate constants without the need for a reference region since it can “borrow” information across the sample and thereby stabilise estimation of otherwise poorly identified parameters. This serves to obviate the need to rely on the imperfect cerebellar reference region to calculate these outcomes, and in simulated data we have previously shown that this approach yields outcomes with a high degree of accuracy at the individual level (Matheson & Ogden, 2022). For instance, we have previously shown in simulated [C11]WAY100635 data that this approach is able to reduce the error in estimates of$BP_{\text{ND}}$and non-displaceable distribution volume ($V_{\text{ND}}$) by over 80% (Matheson & Ogden, 2022). Importantly, owing to the multivariate analysis strategy employed by SiMBA and PuMBA, these methods can exploit multivariate relationships among estimated pharmacokinetic (PK) parameters, which provides inferential advantages over conventional univariate analysis at the group level, including greatly improved statistical power without any corresponding increase in the false positive rate. We therefore anticipated that these methods might yield additional clarity into the study of the 5-HT_1A_R in MDD, and potentially resolve outstanding questions.

In this study, we aimed to apply multivariate analysis strategies, SiMBA and PuMBA, to study potential binding differences between MDD patients and HV, using the combined dataset from all previous studies conducted for which all outcome measures can be estimated, that is, those which were collected at Columbia University. We applied SiMBA to study outcomes derived from a two tissue compartment model (2TCM):$BP_{\text{ND}}$,$BP_{\text{P}}$and$BP_{\text{F}}$, all estimated directly, in contrast to previous studies which used indirect quantification by employing a cerebellar reference region. To allow comparison, since SiMBA has not yet been extended to allow for reference tissue modelling, we use PuMBA to study indirectly-estimated$BP_{\text{ND}}$using the simplified reference tissue model (SRTM) (Lammertsma & Hume, 1996) with the cerebellar white matter as reference region.

## 2 Materials and Methods

### 2.1 Participants and measurement protocols

We considered a sample of 160 individuals, including 57 HV (32 female) and 103 MDD patients (64 female) (Table 1). Of the patients, using the categories defined inParsey et al. (2010), 50 were not-recently-medicated (NRM) (34 female) and 53 were antidepressant-exposed (AE) (30 female). NRM refers to having not received antidepressant medication at any point within the past 4 years, or being antidepressant-naive (Parsey et al., 2010). Importantly, all AE patients were either unmedicated at the time of enrollment or underwent medication washout, and none were on antidepressant medication for at least 2 weeks prior to PET. This sample consists of all participants (Miller et al., 2013;Parsey et al., 2006,^2010^), as well as an additional 6 HV, 5 NRM patients and 12 AE patients who were not included in these studies, but with identical inclusion criteria as well as PET and MRI acquisition protocols. All participants underwent PET examination using the same PET system, with measurements collected for 110 minutes, with 20 frames of duration: 3×$\frac{1}{3}$, 3×1, 3×2, 2×5, 9×10 minutes. All analysis was applied to the same time activity curves (TACs) as were extracted in the original studies, and we did not perform any additional image analysis: the procedures used can be found in the original studies (Miller et al., 2013;Parsey et al., 2006,^2010^).

**Table 1:** Demographic information.

	HV	AE	NRM
Age (years)	38.3 ± 14.3 (18.5 - 70)	39.2 ± 12.1 (18.3 - 70.4)	37.5 ± 13 (18.8 - 63.7)
Sex	32 ♀ 25 ♂	30 ♀ 23 ♂	34 ♀ 16 ♂
Year	2002 (1999 - 2009)	2004 (1999 - 2009)	2006 (1999 - 2009)
f _P_	0.08 ± 0.02 (0.04 - 0.15)	0.07 ± 0.02 (0.03 - 0.15)	0.06 ± 0.02 (0.03 - 0.12)
Injected Dose	7.7 ± 3.5 (2 - 19.8)	7.1 ± 3.3 (1.9 - 18.9)	5.9 ± 2.4 (1.2 - 15.1)
HDRS-24	1.1 ± 1.9 (0 - 9)	24 ± 7 (11 - 41)	25.8 ± 6.7 (14 - 42)
BDI	1.8 ± 2.6 (0 - 10)	26.3 ± 10.7 (5 - 53)	25.5 ± 9.6 (10 - 50)
Suicide Attempt History	0%	39.6%	22%

Mean ± SD (Minimum – Maximum).

### 2.2 PET modelling and analysis

SiMBA and PuMBA are hierarchical, multivariate, multifactor models. SiMBA performs simultaneous quantification of pharmacokinetic parameters and statistical analysis with TACs as input data, while PuMBA is a method for statistical analysis using externally estimated pharmacokinetic parameters. These models are hierarchical because they make use of partial pooling across the many individuals and regions to borrow strength across the dataset: parameters are estimated as originating from a common distribution, and individual estimates are shrunk towards the global mean. For quantification (with SiMBA), this has the effect of improving the identifiability and accuracy of estimated model parameters, thereby stabilising the direct quantification of binding potential, that is, without using a reference region. As multifactor models, they make use of multiple hierarchies at once: individual differences and regional differences are estimated across overlapping combinations of the full dataset. In this way, these models perform a progressive variance decomposition, thereby making these models highly interpretable by automatically separating the influence of different factors, for instance individual differences from regional differences. The methodologies for SiMBA (Matheson & Ogden, 2022) and PuMBA (Matheson & Ogden, 2023) are more fully described in their original publications alongside their performance in simulated data.

When applying the 2TCM (Gunn et al., 2001), we performed quantification and analysis using SiMBA (Matheson & Ogden, 2022). We defined the model with specific binding represented using either$BP_{\text{ND}}$,$BP_{\text{P}}$or$BP_{\text{F}}$, estimated directly, that is, using rate constants. Although this approach has previously been recommended against in conventional pharmacokinetic modelling of PET data using nonlinear least squares estimation (Slifstein & Laruelle, 2001) as it tends to be highly error-prone; we have previously shown that with SiMBA these outcome measures are estimated much more accurately, and with approximately 80% less measurement error (Matheson & Ogden, 2022). For the$BP_{\text{ND}}$model, the PK parameters were the natural logarithms of$K_{\text{1}}$,$V_{\text{ND}}=K_{\text{1}}\text{​}/k_{\text{2}}$,$BP_{\text{ND}}=k_{\text{3}}\text{​}/\text{​}k_{\text{4}}$,$k_{\text{4}}$, and$v_{\text{B}}$. For the$BP_{\text{P}}$model,$BP_{\text{ND}}$above was replaced by$BP_{\text{P}}$=$K_{\text{1}}k_{\text{3}}\text{​}/\text{​}k_{\text{2}}k_{\text{4}}$. For the$BP_{\text{F}}$model, the arterial input function (AIF) was multiplied with the measured$f_{\text{P}}$value to yield an input function representing the concentration of metabolite-corrected free radioligand in arterial plasma. Hence, the parameters of the latter model were the natural logarithms of$K_{\text{1}}\text{​}/\text{​}f_{\text{P}}$,$V_{\text{ND}}\text{​}/\text{​}f_{\text{P}}$,$BP_{\text{F}}=K_{\text{1}}k_{\text{3}}\text{​}/\text{​}k_{\text{2}}k_{\text{4}}f_{\text{P}}$,$k_{\text{4}}$, and$v_{\text{B}}$. In all models described, the$v_{\text{B}}$parameter represents the blood volume fraction, that is, the fraction of the regional volume comprised blood vasculature and whose TAC is represented by the measured whole blood curve. Because whole blood was not measured in this study, we made use of whole plasma as a proxy for whole blood, as performed in previous studies using these data (Miller et al., 2013;Parsey et al., 2006,^2010^). Across all 2TCM SiMBA models, the estimated geometric mean$v_{\text{B}}$using whole plasma was estimated to be 4.4%, which is consistent with the typical assumption of 5%.

When applying SRTM (Lammertsma & Hume, 1996), we were not able to use SiMBA since the reference region-based functionality has not yet been developed. Rather, we made use of PuMBA (Matheson & Ogden, 2023), using the parameters estimated using conventional nonlinear least squares (NLS) estimation, with the cerebellar white matter as reference region. NLS estimation was applied using kinfitr (Matheson, 2019;Tjerkaski et al., 2020). For NLS modelling, weights were defined according to the default kinfitr method, that is, the square root of the product of the frame duration and the mean TAC value, after being un-corrected for radioactive decay. As input parameters for the PuMBA model, we used the natural logarithms of$R_{\text{1}}$,${k^{\prime }}_{2}$and$BP_{\text{ND}}$.

Univariate analysis was performed using linear mixed effects (LME) modelling, in which the natural logarithm of the relevant binding potential (BP) outcome was defined as the dependent variable, with covariates for region, sex, age, a group-by-region interaction term (without an overall group term), as well as a random intercept for individual. The reason for excluding an overall group term was to aid interpretability of the coefficients, as when defined in this way they compare each of the regional group effects with zero, rather than with the average overall group effect. SiMBA and PuMBA analyses were defined with the same set of covariates forBP, but these models additionally require defining regression models for each of the covariates. We defined partial pooled (random effect) deviations for region, individual, and TAC, as previously described (Matheson & Ogden, 2022,^2023^). For the 2TCM, age and sex were defined as fixed effect covariates for the natural logarithm of$K_{\text{1}}$, and for$V_{\text{ND}}$. For PuMBA analysis of the SRTM results, age and sex were defined as fixed effect covariates for the natural logarithm of${k^{\prime }}_{2}$.

For the Bayesian multivariate models, we used moderately informative priors for the global parameter intercepts, as described previously (Matheson & Ogden, 2022,^2023^), and weakly informative regularising priors otherwise. Zero-centred regularising priors were defined over the standard deviation of the partially pooled effects, with standard deviations defined in such a way as to exclude implausible magnitudes of variation. For the differences between MDD patients and HV, we defined zero-centred regularising priors with a normal distribution centred at zero with a standard deviation of 0.2. This implies that our prior beliefs are that no group differences are most likely, and that we are progressively more skeptical of differences of larger magnitude. The prior assigns 18% of its prior probability to differences less than 10%, 64% to differences less than 20%, and 81% to differences less than 30%. The prior is two-sided, meaning that our model assigns only 9% prior probability to increases greater than 30%, and 9% to decreases greater than 30%. The priors are more fully described inSupplementary Materials S2.

All modelling and analysis were performed using R 4.0.5 (Shake and Throw) (R Core Team, 2022). Linear mixed-effects model analysis was performed using lme4 (Bates et al., 2015). Markov Chain Monte Carlo fitting was performed using STAN (Carpenter et al., 2017) together with the brms R package (Bürkner, 2017).

## 3 Results

### 3.1 Group comparisons

#### 3.1.1 Plasma-free fraction

**Fig. 1.**
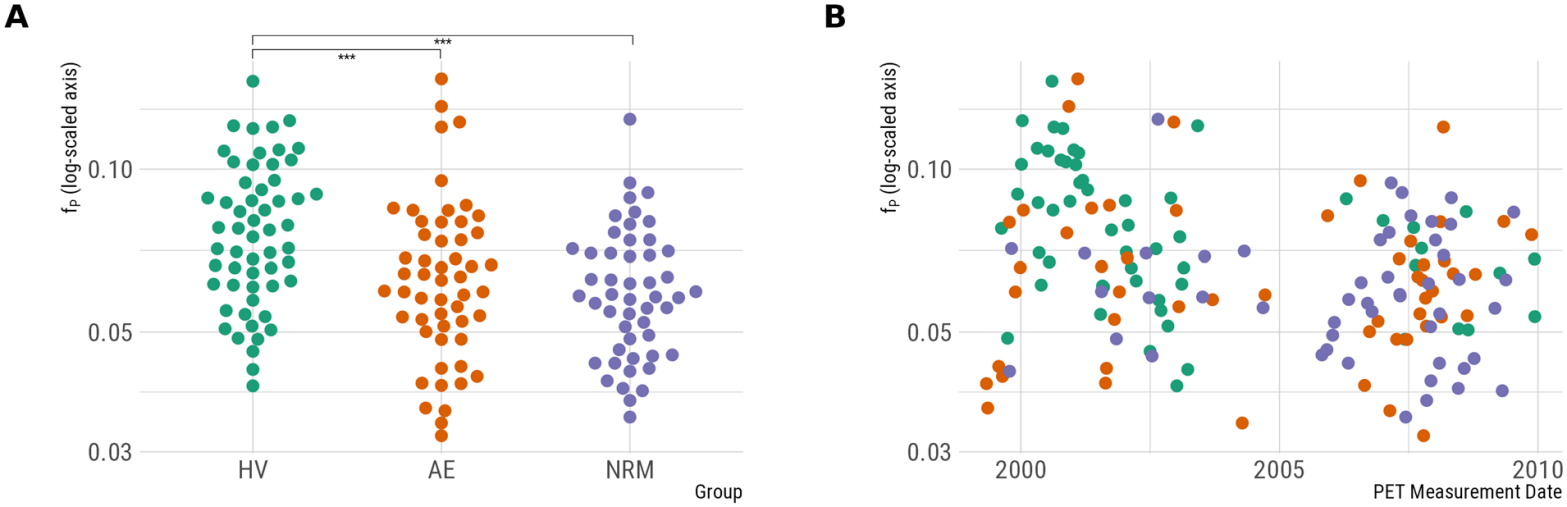
Potential explanatory factors for measured plasma-free fraction values. (A) Group differences in the plasma-free fraction. (B) Plasma-free fraction as a function of the date of PET measurement.

We observed group differences in the natural logarithm of$f_{\text{P}}$($F_{\text{2,157}}$= 10.1, p<0.001) (Fig. 1A), with differences between HV and both AE patients (Estimated difference in$f_{\text{P}}$= 22%, p = 0.003) and NRM patients (Estimated difference in$f_{\text{P}}$= 28%, p<0.001), but no significant differences between the two patient groups (Estimated difference in$f_{\text{P}}$= 6%, p = 0.631). If these differences in measured$f_{\text{P}}$values reflect true differences in the plasma-free fraction, then it is important that they be accounted for during quantification by estimating$BP_{\text{F}}$. However, due to the inhomogeneous distribution of sampling from the different participant groups over the years of data collection, resulting in the majority of HV being imaged before the patient groups, it is also possible that they could be explained by experimental drift in the measurement of$f_{\text{P}}$(Fig. 1B). If this is the case, the$BP_{\text{F}}$estimates will be systematically biased, and global group differences will be artificially induced.

To explore this possibility, we fit an additional analysis with fixed effects for group differences as before, but including a smooth term for the date of PET measurement to describe any potential experimental drift. The model was fitted using the mgcv (Wood, 2011) package using a thin-plate spline regression with 10 degrees of freedom with the smoothing penalty estimated using restricted maximum likelihood estimation. Using this model, the smooth experimental drift term was statistically significant ($F_{\text{8.1}}$= 5.00, p<0.001), while the group differences were no longer significant ($F_{\text{2}}$= 1.225, p = 0.297). This suggests that it is at least plausible that the group differences in$f_{\text{P}}$might be caused by experimental drift, although neither possibility can be conclusively ruled out based on the data at hand because of the inhomogeneous time distribution of the groups.

#### 3.1.2 Univariate analysis

**Fig. 2.**
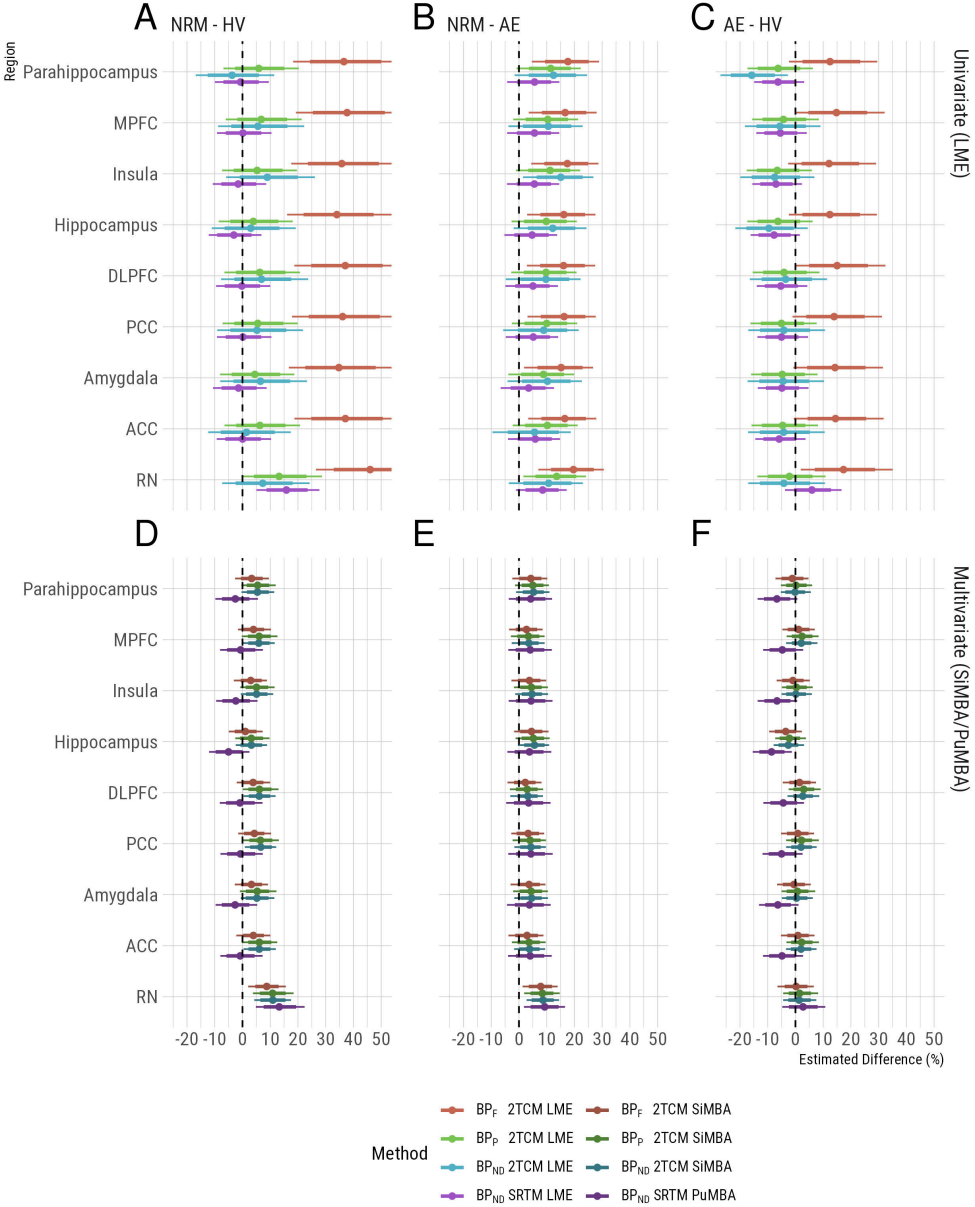
Group differences and their associated uncertainty, presented as percentage differences between groups. Colors represent the outcome measure used, in which lighter colours represent univariate LME results (A-C) and darker colours represent multivariate results (D-F). Uncertainty intervals represent the 80% and 95% confidence/credible intervals with the thicker and thinner bands respectively. Regional abbreviations are as follows: DLPFC is dorsolateral prefrontal cortex, MPFC is medial prefrontal cortex, ACC is anterior cingulate cortex, PCC is posterior cingulate cortex, and RN is the raphe nuclei.

Using conventional univariate LME analysis of PET outcomes, we broadly replicate the results of previous studies (Miller et al., 2013;Parsey et al., 2006,^2010^) and their associated discrepancies between the results obtained using different outcome measures (Fig. 2A–C). In contrast to previous analyses, all 2TCM outcomes are estimated directly from rate constants, as opposed to indirectly using the cerebellum as a reference region, in order to be comparable to the multivariate analyses. Also, in contrast to previous analyses, the ratio of$K_{\text{1}}$/$k_{\text{2}}$in each region is not constrained to be equal to the same ratio in the white matter cerebellum (i.e., a constrained 2TCM was used in previous analyses).

Focusing on the differences between NRM MDD patients and HV (Fig. 2A), which has been the primary focus of previous studies, large global differences are observed between groups using$BP_{\text{F}}$. However, owing to the large differences in$f_{\text{P}}$(Fig. 1A) which may or may not be caused by true biological differences, it is unclear whether these differences in$BP_{\text{F}}$are genuine or a methodological artefact. The results for$BP_{\text{P}}$and$BP_{\text{ND}}$, in which$f_{\text{P}}$differences are not corrected for, do not exhibit the same global differences between groups (Fig. 2A). In previous studies of these data (Miller et al., 2013;Parsey et al., 2006,^2010^), these outcomes have been estimated indirectly, that is, using both arterial plasma as well as a reference tissue, due to concerns that direct estimation may compromise the accuracy of estimated outcomes (Slifstein & Laruelle, 2001). However, the fact that we fully replicated the outcome measure discrepancies observed in previous studies of these data suggests that our use of direct estimation is unlikely to be a major source of differences between this and previous analyses.

Lastly, using indirect estimation of$BP_{\text{ND}}$using SRTM with the cerebellar white matter as a reference region, we observe higher$BP_{\text{ND}}$in NRM patients relative to HV in the raphe nuclei (RN).

#### 3.1.3 Multivariate analysis

Using the multivariate analysis approaches, SiMBA and PuMBA, the uncertainty intervals are much narrower than those calculated using the univariate methods as shown previously (Matheson & Ogden, 2022) and, importantly, the estimates are broadly consistent across all four outcomes (Fig. 2D–F). Focusing on the differences between NRM patients and HV again (Fig. 2D), all outcomes show regionally-specific higher binding potential in the RN of NRM patients relative to HV. NRM patients also show higher RN binding potential compared to AE patients consistently across outcome measures (Fig. 2E). These similarities between outcomes are observed in spite of the differences in$f_{\text{P}}$between groups which would affect the estimation of$BP_{\text{F}}$but not the other outcome measures. Similarly, SRTM$BP_{\text{ND}}$also exhibits a similar pattern of results as the 2TCM outcomes above (Fig. 2D–F), in spite of both being an indirect estimate of$BP_{\text{ND}}$, that is, estimated without the use of arterial blood data; as well as being estimated using multivariate analysis of a different set of PK parameters, that is, those estimated by SRTM.

In all the remaining regions where post-synaptic heteroreceptors are located, there was a tendency for slightly higher binding in NRM patients compared with both HVs and AE patients for the 2TCM outcomes; however, these estimates had a magnitude of approximately half of that observed in the RN, and mostly had 95% credible intervals which overlapped with 0. In order to study the regional specificity further, we fit additional models for each outcome measure with fixed effects for overall group differences in the RN and for the serotonin projection regions, with random slopes for the serotonin projection region (i.e., allowing variation in regional differences around their overall mean). Comparing the magnitude of group differences in the RN with the mean difference in projection regions, we show that differences are larger in the RN (P[$\hat{\beta }>$0] = 99.5% for 2TCM$BP_{\text{F}}$, 98.9% for 2TCM$BP_{\text{P}}$, 99.3% for 2TCM$BP_{\text{ND}}$and 99.5% for SRTM$BP_{\text{ND}}$) comparing NRM patients with controls. However, BP is still higher on average in projection regions in NRM patients compared to controls for 2TC models (although the 95% credible interval partially overlaps with 0 for$BP_{\text{F}}$): (P[$\hat{\beta }>$0] = 91.4% for$BP_{\text{F}}$, 97.7% for$BP_{\text{P}}$, and 98.5% for$BP_{\text{ND}}$). Within the projection regions, there is minimal regional variation in the magnitude of differences, that is, the random slopes: the standard deviation ofΔlogBP < 0.01 for all outcome measures for both NRM–control comparisons as well as AE–control comparisons. All estimates and directional probabilities comparing regional differences as both fixed effects and random slopes are provided inSupplementary Materials S3.

Notably, the pattern of results for the projection regions was inconsistent between direct (2TCM) and indirect (SRTM) quantification approaches. For the NRM–HV comparisons, all direct approaches demonstrate elevations in binding potential in NRM patients compared with HV for projection regions, while the indirect SRTM approach does not (P[$\hat{\beta }>$0] = 18.7%). Similarly, for the AE–HV comparisons (Fig. 2F), all direct quantification approaches are consistent with no differences, yet the SRTM indirect quantification indicates lower binding potential in AE patients in projection regions (P[$\hat{\beta }<$0] = 97.4%). One possible explanation for these inconsistencies could be that cerebellar white matter binding is higher in MDD compared with HV. WhileParsey et al. (2010)showed that$V_{\text{T}}$in cerebellar grey matter was elevated in MDD groups compared to HV, they did not observe statistically significant differences in cerebellar white matter. To explore this possibility, we fit an additional exploratory SiMBA model examining group differences in$BP_{\text{F}}$where cerebellar grey and white matter regions were additionally included as regions of interest. This model indicated that$BP_{\text{F}}$was higher in MDD, regardless of antidepressant exposure, compared with HV in both cerebellar grey matter (AE - HV: 22% [12 - 32%] 95% CI, P[$\hat{\beta }>$0]>99.9%; NRM - HV: 39% [28 - 50%] 95% CI, P[$\hat{\beta }>$0]>99.9%) and cerebellar white matter (AE - HV: 9% [0.2 - 20%] 95% CI, P[$\hat{\beta }>$0] = 97.8%; NRM - HV: 13% [4 - 23%] 95% CI, P[$\hat{\beta }>$0] = 99.6%). This finding supports the hypothesis proposed byParsey et al. (2010)that the small degree of specific binding in cerebellar grey matter is greater in MDD; and that this may also be the case for cerebellar white matter, which might, therefore, explain the bias observed in the SRTM results above.

Lastly, we did not observe the associations between sex and binding which have been reported in prior studies (Parsey et al., 2006,^2010^). However, we did observe sex differences in$K_{\text{1}}$, with males showing a lower rate of transfer from arterial plasma to tissue compared with females. This outcome was reasonably consistent across all 2TC models examining$BP_{\text{F}}$(-5.1%, P[$\hat{\beta }<$0] = 97.3%),$BP_{\text{P}}$(-5.6%, P[$\hat{\beta }<$0] = 97.9%), and$BP_{\text{ND}}$(-5.9%, P[$\hat{\beta }<$0] = 97.3%).

### 3.2 Individual-level estimate associations

Given the consistency of the group-level inferences between outcome measures despite group differences in$f_{\text{P}}$, we sought to compare individual-level outcomes. To this end, we extracted the partially pooled individual-level estimates (i.e.,$\tau_{BP_{\text{ND}}\left[j\right]}$inMatheson & Ogden, 2022), also known as the random effects, for the binding potential outcomes. These figures represent the mean individual binding potential values after adjustment for all of the included covariates (including group membership) as well as regional differences. This allows us to examine the consistency of individual binding potential estimates across outcomes. We extracted the individual-level binding potential estimates for SiMBA with$BP_{\text{F}}$, SiMBA with$BP_{\text{ND}}$, and for PuMBA with SRTM$BP_{\text{ND}}$.

**Fig. 3.**
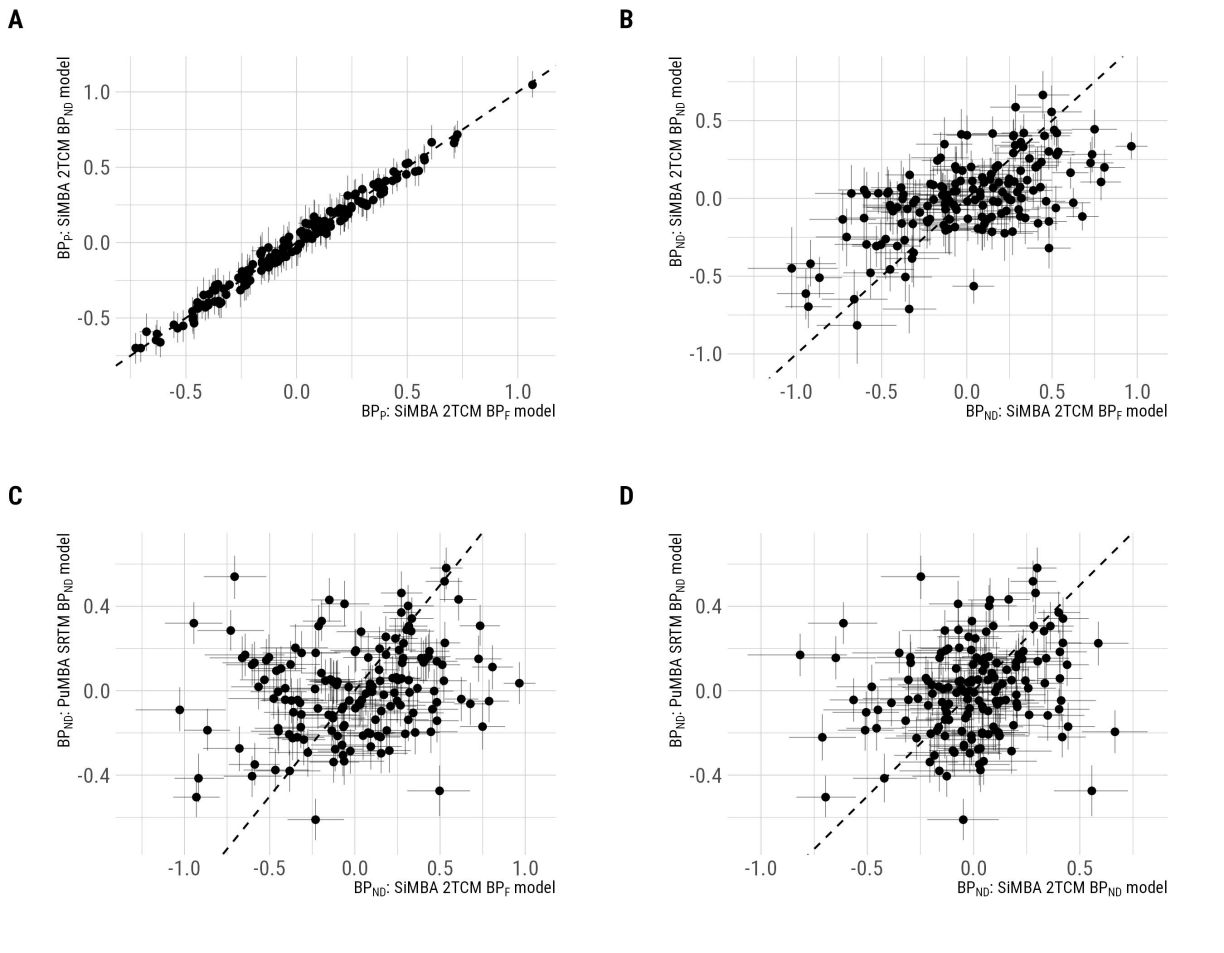
Individual-level binding potential estimates compared between models, and lines of identity. (A) Comparisons of$BP_{\text{P}}$estimated by SiMBA with and without the correction of the AIF for$f_{\text{P}}$. (B) Comparisons of$BP_{\text{ND}}$estimated by SiMBA with and without the correction of the AIF for$f_{\text{P}}$. (C) Comparison of$BP_{\text{ND}}$estimated by SiMBA with correction of the AIF for$f_{\text{P}}$and indirect estimates calculated using PuMBA with SRTM. (D) Comparison of$BP_{\text{ND}}$estimated by SiMBA without correction of the AIF for$f_{\text{P}}$and indirect estimates calculated using PuMBA with SRTM.

Comparing direct estimates, we observe a high degree of correspondence (r>0.99) between$BP_{\text{P}}$values estimated by the 2TCM with and without correction for$f_{\text{P}}$(Fig. 3A). In contrast, we observe weaker associations (r = 0.59) between$BP_{\text{ND}}$values estimated by these two models (Fig. 3B). Keeping in mind that$BP_{\text{P}}$is equal to the product of$BP_{\text{ND}}$and$V_{\text{ND}}$, this implies that correction of the AIF for$f_{\text{P}}$differences alters the balance of$BP_{\text{ND}}$and$V_{\text{ND}}$estimates at the individual level, but that changes in each one of these parameters are accommodated for by corresponding changes in the other within the SiMBA model. When instead examining the correspondence between direct (arterial-blood based 2TCM) and indirect (reference-region-based SRTM) estimates of$BP_{\text{ND}}$, we see little to no association between estimates either with (r = 0.23,Fig. 3C) or without (r = 0.19,Fig. 3D) correction for$f_{\text{P}}$.

### 3.3 Correlation matrices

Both SiMBA and PuMBA models exploit the multivariate associations between estimated PK parameters to improve inferential power. One of the assumptions of these models is that the (random effect) correlations between PK parameters are shared across the total sample after correction, and that they do not differ between groups. The model is defined in such a way that it should be robust to small deviations from this assumption, as this residual variance can be accommodated by Individual×Region (i.e., TAC) variance; however, large deviations between groups could potentially be problematic. To test this assumption, we fit an SiMBA$BP_{\text{ND}}$model to each of the three subgroups independently, and extracted the estimated individual-level correlation matrices for comparison. We selected the SiMBA$BP_{\text{ND}}$model, as$BP_{\text{ND}}$is less stable than$BP_{\text{P}}$, and ought to exaggerate any potential differences between groups, especially considering the relatively smaller sample sizes of each separate group. The results are shown inFigure 4. Based on the high degree of similarity of these matrices, we do not anticipate that this is a source of appreciable bias in the results of our models.

**Fig. 4.**
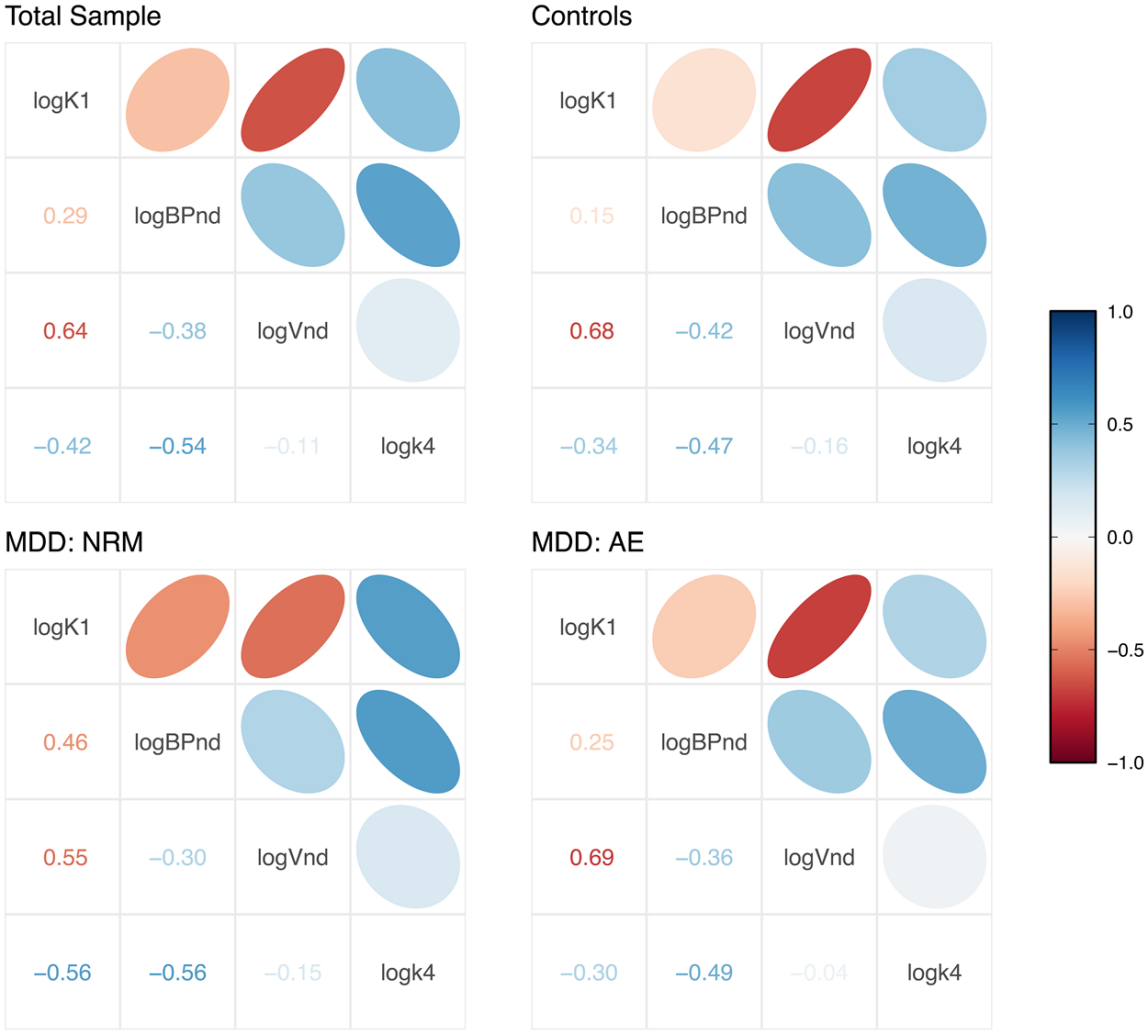
Individual-level random effect correlation matrices were estimated for the total sample as well as each patient subgroup independently. The similarity of these matrices suggests both that their estimation is robust, but also that there do not appear to be any clear systematic differences between groups that are not more likely to be explained by estimation inaccuracies.

## 4 Discussion

We have applied novel hierarchical multivariate analysis methods, that we have previously shown to improve quantitative accuracy and inferential efficiency (Matheson & Ogden, 2022,^2023^), to the largest-to-date sample of [C11]WAY100635 PET data, including full arterial sampling and measurement of the radioligand$f_{\text{P}}$, to study differences between HV and participants with MDD. In contrast to previous studies, using these new methodological approaches we find highly consistent results using$BP_{\text{F}}$,$BP_{\text{P}}$and$BP_{\text{ND}}$, and using both the 2TCM and SRTM pharmacokinetic models. Concordance across binding outcome measures and models is expected in theory, but has not been observed in previous studies employing traditional PET analysis methods to study the 5-HT_1A_receptor in MDD. Moreover, while previous studies found generalised increases across the brain in not-recently-medicated MDD patients relative to controls, which were restricted to$BP_{\text{F}}$, our current study is novel in that it both corroborates these findings across all outcome measures, as well as extending these findings by showing that these differences are most pronounced in the RN. This regional specificity, however, is consistent with the particular neurobiological role played by 5-HT_1A_autoreceptors in the RN relative to heteroreceptors in the rest of the brain. This study may, therefore, help resolve the longstanding discrepancies observed in studies employing PET imaging to quantify the 5-HT_1A_R binding in MDD.

The regional specificity of our findings is of particular interest given the particular role of the 5-HT_1A_R in the RN, where it functions as a somatodendritic autoreceptor, as opposed to its role as a heteroreceptor in the rest of the brain. These autoreceptors control serotonin neuron firing and serotonin release throughout the brain through hyperpolarising the serotonin neurons via a potassium channel in response to increased extracellular serotonin concentrations (Barnes & Sharp, 1999;Hoyer et al., 2002;Montalbano et al., 2015). It has long been speculated based on rodent studies (Blier & De Montigny, 1983;Gardier et al., 1996;Piñeyro & Blier, 1999) that 5-HT_1A_autoreceptors mediate the delayed onset of action of drugs which increase serotonin levels, such as selective serotonin reuptake inhibitors (SSRIs). Initially, increasing extracellular serotonin concentrations results in 5-HT_1A_autoreceptor-induced hyperpolarisation of serotonin neurons, and inhibition of firing and serotonin release. With prolonged treatment, these receptors become desensitised and eventually downregulated through internalisation, resulting in enhanced firing and serotonin release and emergence of an antidepressant effect (Blier & De Montigny, 1983;Gardier et al., 1996;Piñeyro & Blier, 1999;Richardson-Jones et al., 2010), leading to a gradual increase in treatment effects over several weeks (Taylor et al., 2006). In mice genetically engineered to specifically express high or low levels of 5-HT_1A_autoreceptors, the mice with low levels show less depression-like behaviour, while also exhibiting greater antidepressant response to SSRI medication (Richardson-Jones et al., 2010). Our findings are consistent with these results: we observe higher 5-HT_1A_autoreceptor binding in not-recently-medicated patients compared to both HV, suggesting the possibility of its role in the pathophysiology of MDD, as well as compared to antidepressant-exposed MDD patients, suggesting the possibility of residual treatment effects downregulating 5-HT_1A_R binding in the AE group as shown inGray et al. (2013).

The question remains why this pattern of results was not observed in previous studies: if binding potential for [C11]WAY100635 in the RN is higher in unmedicated MDD, then why have results been so inconsistent? For$BP_{\text{P}}$in particular, results have been inconclusive, despite its being less sensitive to radiometabolite contamination than$BP_{\text{ND}}$, and less sensitive to any potential methodological biases in the measurement of$f_{\text{P}}$than$BP_{\text{F}}$. We offer two potential answers to this question. Firstly, these differences were observed in the RN: this region is very small and its TACs have higher measurement error compared to the other brain regions examined here. For this reason, its estimation is more susceptible to quantification inaccuracies than the other regions. SiMBA and PuMBA both make use of hierarchical modelling across both individuals and brain regions, which allows them to effectively borrow strength from the rest of the dataset to improve inferences made. For SiMBA in particular, quantification is performed within the model, and using the rest of the data, resulting in substantial improvements to the accuracy of quantification at the individual level, with greatest improvements observed for the RN (Matheson & Ogden, 2022). Secondly, previous studies have made use of univariate analysis methods to study this research question. This means that binding potential values are estimated for each region of each individual and entered into a statistical test, while all the other PK parameters estimated by the model are discarded. We have shown for both SiMBA (Matheson & Ogden, 2022) and PuMBA (Matheson & Ogden, 2023) that there is additional information within these discarded parameters and their covariance structures, which can be exploited to improve, sometimes dramatically, the power of group-level inferences made using binding potential—particularly with larger sample sizes. In simulations of [C11]WAY100635 data, using$BP_{\text{P}}$as an outcome measure, multivariate inferential power was equivalent to that of a sample approximately twice the size using univariate analysis (Matheson & Ogden, 2022). To this end, as part of this study, we performed a power analysis to estimate sample size required to test the differences observed in this study in$BP_{\text{P}}$between NRM patients and HV in the RN for$BP_{\text{P}}$using a univariatet-test. The results of the power analysis are reported inFigure 5, showing that a sample size of 158 individuals (95% CI: 57–1176) would be required in*each group*to achieve 80% power with conventional univariatet-test, owing to the magnitude of group differences observed which correspond to a Cohen’sdof 0.32 (95% CI: 0.11–0.53). This is usually interpreted as a small effect size: to contextualise the magnitude of these differences, this figure implies that selecting a healthy control and an NRM MDD patient at random, the probability of the NRM patient having a higher binding potential than the control is only 58.7% (95% CI: 54.4%–64.6%) due to the substantial degree of overlap between groups (Supplementary Materials S4). Hence, even including all participants from all previous studies of this research question (Shrestha et al., 2012;Wang et al., 2016) in a singlet-test, even after correcting for centre differences, would be underpowered to detect such a difference. This small effect size refers to population differences, and may be so small because of a combination of factors, such as clinical heterogeneity (e.g., 5-HT_1A_autoreceptors are greatly increased in some patients, but not in others), population heterogeneity (e.g., 5-HT_1A_R levels may differ a great deal between individuals due to different genetics or environment), or because many biological systems are affected, of which the 5-HT_1A_R is only one component. Regardless of its cause, while these differences may be informative from a scientific perspective, their clinical utility is most likely limited.

**Fig. 5.**
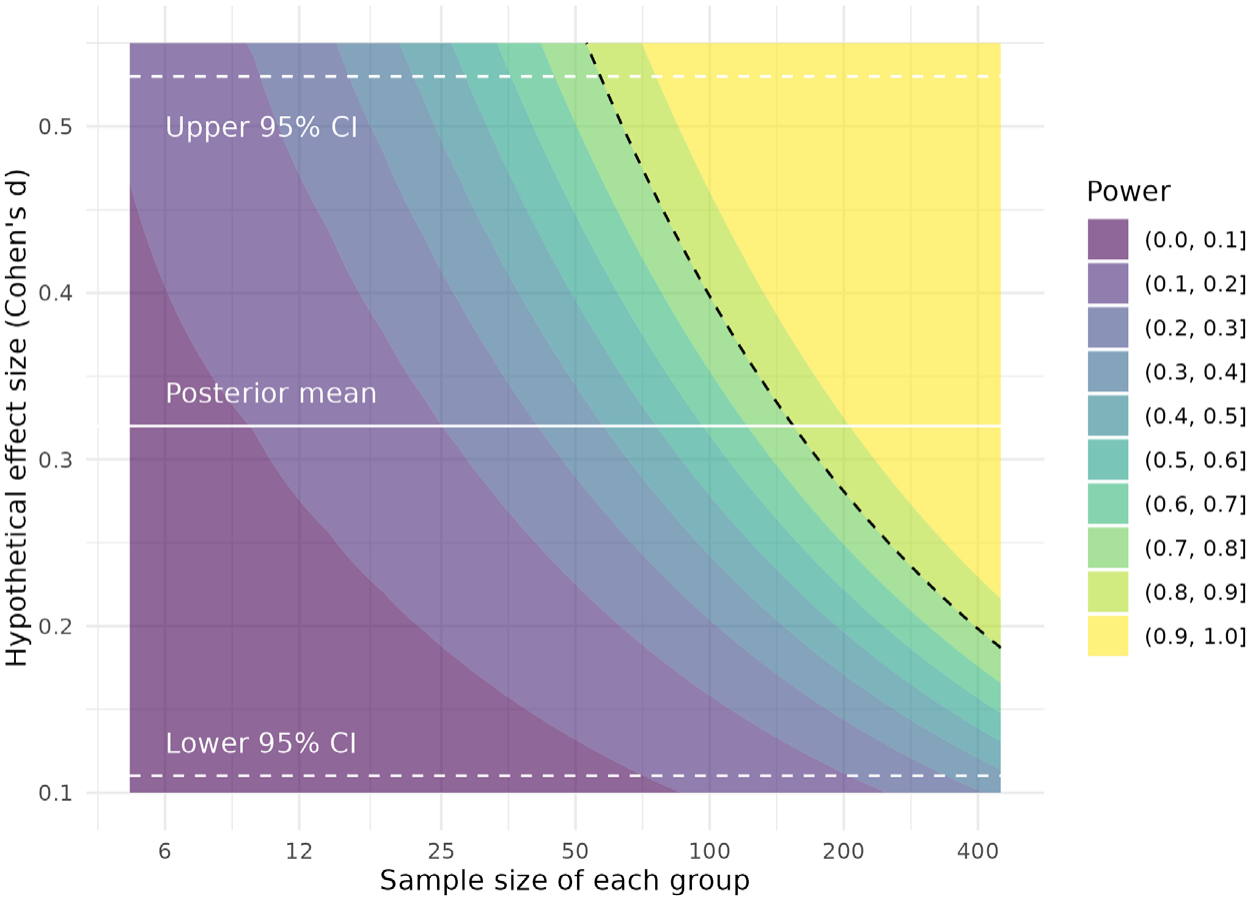
Based on the results of our analysis, the required samples sizes for a univariatet-test comparing BP_ND_between HV and not-recently medicated MDD are shown as a function of the hypothetical true effect size. The dotted black line shows where 80% power lies.

One surprising outcome was the overall similarity of the group-level inferences across the different binding potential outcomes in spite of the dissimilarity of the individual-level binding potential estimates, also known as the random effect estimates. This shows that the hierarchical parameters and their shrinkage were different for each of these outcome parameters, mirroring the variability of previous results across different outcome parameters. However, by acknowledging and exploiting the multivariate covariance structure in the data between all PK parameters simultaneously, our models were able to make inferences which were more resilient to these differences. This raises the important issue of how stable these multivariate relationships really are: as it is implemented, the model requires that the correlation structure is reasonably consistent between groups. To this end, by fitting the model to each subgroup of the data independently and showing that the estimated correlation structure was highly similar between groups (Fig. 4), we conclude that this is unlikely to have been a cause for concern.

Delving more deeply into the correlations between different binding potential outcomes, we further examined the associations between outcomes estimated using both SiMBA and NLS in the raphe nuclei (Supplementary Materials S5). Despite the moderate associations between different binding potential estimates, we show that correlations between SiMBA and NLS estimates were high (r≥0.94) for both$BP_{\text{P}}$and$BP_{\text{F}}$. Correlations were lower for$BP_{\text{ND}}$(r = 0.42), although this could merely be caused by the inaccuracy of NLS estimates. This is supported by the fact that correlations between$BP_{\text{ND}}$and both$BP_{\text{P}}$and$BP_{\text{F}}$were higher for SiMBA than for NLS in every region (Supplementary Materials S6). Together, these results lend support to our proposal that while SiMBA is likely more accurate in its parameter estimates (as shown in simulated data inMatheson & Ogden, 2022), the consistency between outcome measures is likely to result from the additional stability afforded by the multivariate shrinkage utilised by these models.

The most clear differences in the results were between outcomes using direct and indirect means. Not only were there differences in group-level inferences, but the individual-level$BP_{\text{ND}}$estimates were also uncorrelated. The problems with indirect estimation of$BP_{\text{ND}}$for [C11]WAY100635 have been described previously (Osman et al., 1998;Parsey et al., 2005,^2010^): owing to the low distribution volume of the cerebellar grey and white matter (and the low$V_{\text{ND}}$of [C11]WAY100635 more generally), the small absolute deviations in the distribution volume caused by contamination by small concentrations of radioactive metabolites constitute large proportional deviations. This, therefore, exerts an outsized influence on$BP_{\text{ND}}$compared to, for instance,$BP_{\text{P}}$. However, the use of indirect estimation of$BP_{\text{ND}}$for [C11]WAY100635 with SRTM is not limited to this issue. The SRTM model makes four primary assumptions (Lammertsma & Hume, 1996;Salinas et al., 2014). Firstly, the model assumes that there should be no displaceable component in the reference region. This is, however, not true of the cerebellar grey matter (Hirvonen et al., 2007;Parsey et al., 2005,^2010^), and potentially of the white matter to a smaller extent due to spill-in from partial volume effects. Secondly, SRTM assumes that the blood volume to the tissues is negligible: given that uptake is so low in the cerebellum, this assumption is unlikely to be met. Thirdly, SRTM assumes that there are one-tissue compartment model (1TCM) kinetics in both the target and reference regions, which is not met, as the 1TCM does not provide an adequate fit in either. Lastly, SRTM makes the assumption that$V_{\text{ND}}$is the same in all regions. Using SiMBA, however, the model evaluates variation in regional mean$V_{\text{ND}}$estimates (i.e.,$\upsilon_{V_{\text{ND}}}$) for the 2TC models (Matheson & Ogden, 2022). From the model including the cerebellar regions,$V_{\text{ND}}$is estimated to be lowest compared to the mean in the cerebellar white matter (-35% 95% CI: -42.6% - -28.1%), followed by the raphe nuclei (-16.5% 95% CI: -29.9% - -6.1%) and the cerebellar grey matter (-11.7% 95% CI: -23.5% - -1.3%), while all the other regions had credible intervals which overlapped with the mean value. This provides evidence that this assumption may also not be fulfilled. In sum, there is reason to believe that none of the four primary assumptions of SRTM are fulfilled for [C11]WAY100635. For this reason, as well as those discussed inShrestha et al. (2012), we consider the outcomes obtained using 2TCM to be more reliable than those of SRTM; however, the consistency of its inferences to those of the 2TCM showing most pronounced differences in the RN is interesting nonetheless. Similarly, we stress that retrospective analyses which treat results obtained using reference tissue modelling, particularly using cerebellar grey matter as a reference region, as equivalent to those obtained using the 2TCM should be interpreted with caution, and recognising the inherent limitations of this outcome and its sources of bias for [C11]WAY100635 PET.

Unfortunately, most of the studies of MDD using [C11]WAY100635 have been performed using reference tissue modelling. This is because this approach does not require arterial sampling, which is costly, technically complex, as well as uncomfortable for participants. Aside from the data included in this study, there exist only two other published PET studies examining [C11]WAY100635 in MDD in which arterial plasma data were collected:Hirvonen et al. (2008)included 21 patients, andMeltzer et al. (2004)included 17 patients. Neither of these two studies, however, collected$f_{\text{P}}$data which would have allowed calculation of$BP_{\text{F}}$. Hence, the data included in this study constitute the entirety of PET data examining MDD with [C11]WAY100635 for which all outcome measures can be estimated. This was discussed inShrestha et al. (2012)which concludes, owing to the$f_{\text{P}}$differences observed inParsey et al. (2006,2010), that$BP_{\text{F}}$is the only outcome measure which can be used to directly study this question because, if there are true differences in$f_{\text{P}}$between groups, then any analysis examining$BP_{\text{P}}$in which these differences are not corrected for will provide biased results. This leaves$BP_{\text{ND}}$, but as discussed earlier, this outcome measure is compromised using reference tissue modelling, and its estimation using the 2TCM is considered too unreliable using conventional methods. For this reason, the present analysis is performed using the sum total of the data for which it is possible to examine these outcome measure discrepancies even though they were all collected at only one PET centre. However, given the similarities of outcomes across outcome measures observed here, this implies that reanalysis of the other datasets for which arterial sampling was performed might also be valuable—even if$f_{\text{P}}$data were not collected.

Notably, despite the large, and statistically significant, group differences in$f_{\text{P}}$, its influence on our inferences about group differences in binding potential appears to have been minimal using SiMBA. FromFigure 3A, we can see that although individual estimates of$BP_{\text{P}}$in SiMBA models are highly correlated with and without correction of the AIF by$f_{\text{P}}$, they are not identical. This is because the SiMBA model performs hierarchical shrinkage, or regularisation, of parameter estimates resulting in slightly different estimates when$f_{\text{P}}$is incorporated into the model. In contrast, using conventional modelling with no shrinkage, the estimates would have been perfectly associated with one another. Because SiMBA makes use of*multivariate*shrinkage in particular, it is able to make use of the covariance structure between all of the estimated PK parameters to constrain its estimation more effectively compared with univariate shrinkage. The fact that the group differences in$f_{\text{P}}$waned in the context of the multivariate hierarchical model does provide indirect support for the hypothesis that these differences may, in part, be caused by methodological drift as opposed to true biological differences. This would have been especially problematic due to the control groups being mostly overlapping betweenParsey et al. (2010,2006)andMiller et al. (2013). This speaks to the importance of recruiting patient and control groups concurrently and homogeneously over time to avoid the potential for such complications, where it becomes difficult to disentangle biological factors from methodological drift.

The present results might be interpreted to imply that correction for$f_{\text{P}}$is unnecessary; however, we would not espouse this position. As discussed above, based on the results of this data alone, we cannot know whether the observed group differences are true or due to methodological factors. The reason for adjusting the AIF for$f_{\text{P}}$is that radioligands, like drugs, bind to plasma proteins, establishing a binding equilibrium, and that only the free fraction of the radioligand not bound to proteins can cross the blood-brain barrier to reach the target of interest. If there are true differences in$f_{\text{P}}$between groups caused by some biological difference, then it is essential that these be corrected for.

Measurement of$f_{\text{P}}$is most commonly performed using one or more arterial plasma samples extracted from the subject prior to radioligand injection, and adding radiolabelled compound before assaying the protein-free fraction using ultrafiltration (Gandelman et al., 1994). In this study, we incorporated measured$f_{\text{P}}$values into our SiMBA model as a scalar quantity, by adjusting the AIF accordingly. While this is consistent with the conventional approach of correcting the estimated$BP_{\text{P}}$value by a scalar$f_{\text{P}}$value, it does not allow for any potential*in vivo*dynamics of$f_{\text{P}}$over time. Using ultrafiltration, as was performed in this study, it is not possible to measure changes in blood$f_{\text{P}}$over the first minutes of the scan due to its long analysis time. On the other hand, it has been reported that equilibrium concentrations of plasma protein binding may even be reached within milliseconds (Peletier et al., 2009;Smith et al., 2010), in which case$f_{\text{P}}$can be considered effectively stable. We, therefore, believe that this approach constitutes a sufficiently good approximation for the data-generating process in order to accommodate differences in$f_{\text{P}}$values at equilibrium; which is indirectly supported by the similarity of our inferences between outcome measures.

In summary, in this study we show that multivariate analysis methods may resolve the longstanding debate over the results of studies of [C11]WAY100635 in MDD, and suggest that NRM patients have higher 5-HT_1A_R binding in the RN compared to both HV and AE patients, although these differences are rather small. We propose that these methods have broad applicability for answering other research questions for which*in vivo*PET imaging is used to study psychiatric, neurological, or even pharmacological questions.

## Supplementary Material

Supplementary Material

## Data Availability

The analysis code used here is based on the R and STAN shared previously from our previous methodological paper presenting this approach (https://github.com/mathesong/SiMBA_Materials) and applied to new data. The specific code used to analyse the current data can be shared upon reasonable request after removal of sensitive patient information. The majority of the current dataset is re-analysed from previous studies, including all participants fromParsey et al. (2006,2010)andMiller et al. (2013). Data from an additional 23 participants were also collected and processed in the same manner but not included in the previous studies.
