# Supplementary material for: PET imaging of the serotonin 1A receptor in major depressive disorder: Hierarchical multivariate analysis of [ 11 C]WAY100635 overcomes outcome measure discrepancies
Source: Imaging Neurosci (Camb). 2024 Oct 25;2:imag-2-00328. doi: 10.1162/imag_a_00328 (PMC12290790; doi:10.1162/imag_a_00328)
Supplement: Supplementary Material [file imag_a_00328-supp.pdf]

Supplementary Materials: PET Imaging of the Serotonin 1A Receptor in Major Depressive Disorder

Granville J. Matheson,[1,2,3,4*] Francesca Zanderigo,[1,3] Jeffrey M. Miller[1,3]
Elizabeth A. Bartlett,[1,3] J. John Mann,[1,3] R. Todd Ogden[1,2,3]

[1]Department of Psychiatry, Columbia University, New York, 10032 NY, USA

[1]Department of Psychiatry, Columbia University, New York, 10032 NY, USA

[2]Department of Biostatistics, Columbia University Mailman School of Public Health, New York, 10032 NY, USA

[3]Molecular Imaging and Neuropathology Area, New York State Psychiatric Institute, New York, 10032 NY, USA

[4]Department of Clinical Neuroscience, Center for Psychiatry Research, Karolinska Institutet and Stockholm County Council, Stockholm, SE-171 76, Sweden

*Correspondence: granville.matheson@ki.se

October 4, 2024

1 Supplementary Materials

1.1 Supplementary Materials S1: PET Quantification Information

PET quantification involves fitting pharmacokinetic (PK) models to the time course of measured radioactivity concentrations in the brain, termed time activity curves (TACs). Using these models, PET allows for quantification of various binding potentials (BP), i.e. the equilibrium concentration of radioligand specifically bound to the target protein, normalized to a reference concentration. While *in vitro* studies can measure the concentration of the target protein available to bind the radioligand, B_{avail}, this is not

possible from a single *in vivo* PET examination. Rather, PET researchers make use of binding potential as a measure of specific binding, which is equal to $B_{\text{avail}}/K_{\text{D}}$, where K_{D} represents the affinity of the target for the radioligand. Hence, as long as K_{D} is unchanged, then BP should be proportional to B_{avail} and can be used as an effective surrogate. However, in order to calculate binding potential, we do so using a suitable reference quantity. As such, there are several different types of binding potential which differ from one another as a function of which reference quantity is used for their calculation, i.e. BP_{X} where X describes the reference quantity.

BP_{F} represents the gold standard PET outcome measure which is, in the absence of active transport, equal to $B_{\text{avail}}/K_{\text{D}}$. The estimation of BP_{F}, however, requires measurement both of the metabolite-corrected radioactivity in arterial plasma during the PET measurement, as well as the protein-free fraction of the radioligand in arterial plasma, f_{P} (Innis et al., 2007; Parsey et al., 2010). Measurement of f_{P} tends to be notoriously prone to measurement error due both to estimation inaccuracy, and especially so when f_{P} values are low as is the case for [^{11}C]WAY100635. Moreover, measurement of f_{P} tends to be sensitive to small experimental biases such as the temperature or pH of plasma samples, the latter of which can even vary as a result of storage time (Hinderling & Hartmann, 2005; Kochansky et al., 2008). Hence, even if data show a reasonably high degree of consistency within individuals, measured f_{P} values could feasibly be impacted by unexpected experimental confounds over time. Measured values can therefore suffer from both variance and bias. On the other hand, BP_{P} which is equal to $f_{\text{P}}B_{\text{avail}}/K_{\text{D}}$, does not require the measurement of f_{P} - however it is sensitive to differences in plasma protein binding between individuals. For this reason, group differences in f_{P} values will yield biased inferences when analysing BP_{P}. Finally, BP_{ND} is one of the most common outcome measures in PET imaging, equal to $f_{\text{ND}}B_{\text{avail}}/K_{\text{D}}$, where f_{ND} represents the radioligand free fraction in the non-displaceable compartment. However, for this reason, group differences in f_{ND}, will also yield biased inferences when analysing BP_{ND}. These binding potential outcomes can be calculated directly from the rate constants of the PK model, which is known as direct estimation, however the reliability of these estimates has been found to be poor in many cases, particularly for BP_{ND} (Slifstein & Laruelle, 2001). Alternatively, binding potential outcomes can be calculated relative to a reference region in the brain which is devoid of specific binding, when one is available, under the assumption that non-displaceable binding is equal throughout the brain. This is known as indirect estimation, and can greatly improve the reliability of binding potential estimates (Slifstein & Laruelle, 2001). Indirect estimation of BP_{ND} is especially common as it does not require collection of arterial plasma samples, which is highly desirable as this procedure is costly, can be uncomfortable for research participants, and adds to the complexity of PET acquisition (Gunn et al., 2001). However, the improvements in reliability using indirect estimation come at a cost, since group differences in reference tissue binding will bias inferences. In summary, all binding potential

outcomes are measures of specific binding relative to a reference concentration and should therefore be approximately proportional to one another in theory; however this is more complicated in practice as estimation inaccuracies or individual differences in f_{P}, f_{ND} or reference-region binding can introduce bias for inference.

1.2 Supplementary Materials S2: Priors

1.2.1 Priors for the SiMBA 2TC models

For all priors, we use the same terminology used in Matheson and Ogden, 2022.

Global Intercepts

Note that all priors are defined over the natural logarithms of the parameters, where the mean is shown first and the standard deviation second.

For the SiMBA BP_{F} model, we used the following priors.

$$\begin{aligned}
\alpha_{K_1} &\sim \mathrm{Normal}(\ \ \ 0.0\ ,\ 0.5) \\
\alpha_{V_{\mathsf{ND}}} &\sim \mathrm{Normal}(\ \ \ 1.5\ ,\ 0.5) \\
\alpha_{BP_{\mathsf{F}}} &\sim \mathrm{Normal}(\ \ \ 3.5\ ,\ 0.5) \\
\alpha_{k_4} &\sim \mathrm{Normal}(-4.0\ ,\ 0.5) \\
\alpha_{\mathsf{v_B}} &\sim \mathrm{Normal}(-3.5\ ,\ 0.5) \\
\alpha_{\sigma} &\sim \mathrm{Normal}(-5.0\ ,\ 1.0)
\end{aligned}$$

For the SiMBA BP_{P} and BP_{ND} models, we used the following priors.

$$\alpha_{K_1} \sim \text{Normal}(-2.5\,, 0.25)$$
$$\alpha_{V_{\mathsf{ND}}} \sim \text{Normal}(-1.0\,, 0.25)$$
$$\alpha_{BP_{\mathsf{ND}}} \sim \text{Normal}(\quad 2.0\,, 0.25)$$
$$\alpha_{BP_{\mathsf{P}}} \sim \text{Normal}(\quad 1.0\,,\ 0.25)$$
$$\alpha_{k_4} \sim \text{Normal}(-4.0\,, 0.25)$$
$$\alpha_{\mathsf{v_B}} \sim \text{Normal}(-3.5\,,\ 0.5)$$
$$\alpha_{\sigma} \sim \text{Normal}(-5.0\,,\ 1.0)$$

Henceforth all priors for BP_{F}, BP_{P} and BP_{ND} are all the same. Hence we will refer to each of them simply as BP_{X}.

Individual deviations

Differences between individuals were defined by specifying the primary pharmacokinetic parameters in one variance-covariance matrix, and $\mathsf{v_B}$ and σ on their own.

$$
\begin{bmatrix} \tau_{K_1} \\ \tau_{V_{\mathsf{ND}}} \\ \tau_{BP_{\mathsf{X}}} \\ \tau_{k_4} \end{bmatrix} \sim \text{MVNormal}\left(\begin{bmatrix} 0 \\ 0 \\ 0 \\ 0 \end{bmatrix}, \sum\nolimits_{\mathsf{Subject}} \right)
$$

$$
\Sigma_{\mathsf{Subject}} = \begin{bmatrix} \sigma_{K_1} & 0 & 0 \\ 0 & \ddots & 0 \\ 0 & 0 & \sigma_{k_4} \end{bmatrix} \mathbf{R}_{\mathsf{Subject}} \begin{bmatrix} \sigma_{K_1} & 0 & 0 \\ 0 & \ddots & 0 \\ 0 & 0 & \sigma_{k_4} \end{bmatrix}
$$

$$
\tau_{\mathsf{v_B}} \sim \text{Normal}(0, \sigma^2_{\mathsf{Subject,v_B}})
$$

$$
\tau_{\sigma} \sim \text{Normal}(0, \sigma^2_{\mathsf{Subject},\sigma})
$$

$$
\begin{aligned}
\sigma_{K_1} &\sim \text{Half-Normal}(0\,,\ 0.3) \\
\sigma_{V_{\mathsf{ND}}} &\sim \text{Half-Normal}(0\,,\ 0.3) \\
\sigma_{BP_{\mathsf{X}}} &\sim \text{Half-Normal}(0\,,\ 0.3) \\
\sigma_{k_4} &\sim \text{Half-Normal}(0\,,\ 0.3) \\
\mathbf{R}_{\mathsf{Subject}} &\sim \text{LKJ}(1)
\end{aligned}
$$

$$
\sigma_{\mathsf{v_B}} \sim \text{Half-Normal}(0\,,\ 0.5)
$$

$$
\sigma_{\sigma} \sim \text{Half-Normal}(0\,,\ 0.3)
$$

Regional deviations

For $\log BP_{\mathsf{X}}$ and $\log K_1$, regional differences were defined as unpooled effects using a dummy (indicator) variable defined with reference to the dorsolateral prefrontal cortex as covariates. For simplicity, all regional differences were defined for all other regions as zero-centred regularising priors with the same SD.

$$
\begin{aligned}
\beta_{K_1} &\sim \text{Normal}(0\,,\ 0.3) \\
\beta_{BP_{\mathsf{X}}} &\sim \text{Normal}(0\,,\ 0.3)
\end{aligned}
$$

For the remaining parameters, regional differences were defined as partially-pooled variables, arising from a common distribution.

$$
\begin{aligned}
\begin{bmatrix} \upsilon_{V_{\mathsf{ND}}} \\ \upsilon_{k_4} \end{bmatrix} &\sim \text{MVNormal}\left(\begin{bmatrix} 0 \\ 0 \end{bmatrix}, \textstyle\sum_{\mathsf{Region}} \right) \\
\Sigma_{\mathsf{Region}} &= \begin{bmatrix} \sigma_{V_{\mathsf{ND}}} & 0 \\ 0 & \sigma_{k_4} \end{bmatrix} \mathbf{R}_{\mathsf{Region}} \begin{bmatrix} \sigma_{V_{\mathsf{ND}}} & 0 \\ 0 & \sigma_{k_4} \end{bmatrix} \\
\upsilon_{\mathsf{v_B}} &\sim \text{Normal}(0, \sigma^2_{\mathsf{Region},\mathsf{v_B}}) \\
\upsilon_{\sigma} &\sim \text{Normal}(0, \sigma^2_{\mathsf{Region},\sigma}) \\
\sigma_{V_{\mathsf{ND}}} &\sim \text{Half-Normal}(0\,,\ 0.1) \\
\sigma_{V_{\mathsf{ND}}} &\sim \text{Half-Normal}(0\,,\ 0.1) \\
\mathbf{R}_{\mathsf{Region}} &\sim \text{LKJ}(2) \\
\sigma_{\mathsf{Region},\mathsf{v_B}} &\sim \text{Half-Normal}(0\,,\ 0.1) \\
\sigma_{\mathsf{Region},\sigma} &\sim \text{Half-Normal}(0\,,\ 0.1)
\end{aligned}
$$

TAC deviations

Residual TAC effects were defined for the major four pharmacokinetic parameters, but were not included for $\mathsf{v_B}$ or σ as these were not considered to be of central importance.

$$\begin{bmatrix}\phi_{K_1}\\ \phi_{V_{\mathsf{ND}}}\\ \phi_{BP_{\mathsf{X}}}\\ \phi_{k_4}\end{bmatrix} \sim \text{MVNormal}\left(\begin{bmatrix}0\\0\\0\\0\end{bmatrix}, \Sigma_{\mathsf{TAC}}\right)$$

$$\Sigma_{\mathsf{TAC}} = \begin{bmatrix}\sigma_{K_1} & 0 & 0\\ 0 & \ddots & 0\\ 0 & 0 & \sigma_{k_4}\end{bmatrix} \mathbf{R}_{\mathsf{TAC}} \begin{bmatrix}\sigma_{K_1} & 0 & 0\\ 0 & \ddots & 0\\ 0 & 0 & \sigma_{k_4}\end{bmatrix}$$

$$\begin{aligned}\sigma_{K_1} &\sim \text{Half-Normal}(0\ ,\ 0.025)\\ \sigma_{V_{\mathsf{ND}}} &\sim \text{Half-Normal}(0\ ,\ 0.025)\\ \sigma_{BP_{\mathsf{X}}} &\sim \text{Half-Normal}(0\ ,\ 0.025)\\ \sigma_{k_4} &\sim \text{Half-Normal}(0\ ,\ 0.025)\\ \mathbf{R}_{\mathsf{TAC}} &\sim \text{LKJ}(2)\end{aligned}$$

Covariates for PK Parameters

For the effects of group on BP, we defined priors for each region and diagnosis separately in order to detect regionally-specific effects.

$$\beta_{\mathsf{Group\ \times\ Region},BP_{\mathsf{X}}} \sim \text{Normal}(\ 0\ ,\ 0.2\ )$$

We also defined priors for age (per decade, centred) and sex to accommodate their influence on the outcomes.

$$
\begin{aligned}
\beta_{\text{Age},K_1} &\sim \text{Normal}(\,0\,,\;0.1\,)\\
\beta_{\text{Age},V_{\text{ND}}} &\sim \text{Normal}(\,0\,,\;0.1\,)\\
\beta_{\text{Age},BP_{\text{X}}} &\sim \text{Normal}(\,0\,,\;0.1\,)
\end{aligned}
$$

$$
\begin{aligned}
\beta_{\text{Sex},K_1} &\sim \text{Normal}(\,0\,,\;0.05\,)\\
\beta_{\text{Sex},V_{\text{ND}}} &\sim \text{Normal}(\,0\,,\;0.05\,)\\
\beta_{\text{Sex},BP_{\text{X}}} &\sim \text{Normal}(\,0\,,\;0.05\,)
\end{aligned}
$$

Measurement Error

For measurement error, σ, we used a combination of a global mean and partially pooled deviations across individuals and regions described above. We also made use of covariates for mean region size, injected radioactivity, and the duration of each frame, as well as a smooth function across the course of the PET measurement to accommodate any residual variability not accounted for by the covariates. The latter smooth term is expected to be more associated with the kinetics of the specific tracer after accounting for the influence of experimental factors.

All covariates are log-transformed and then centred. In this way, the global intercept is still interpretable as the geometric mean of the measurement error, and the covariates are related proportionally to the natural logarithm of the expectation of the measurement error.

$$
\begin{aligned}
\beta_{\text{Region Size},\sigma} &\sim \text{Normal}(\,0\,,\;0.3\,)\\
\beta_{\text{Injected Radioactivity},\sigma} &\sim \text{Normal}(\,0\,,\;0.5\,)\\
\beta_{\text{Frame duration},\sigma} &\sim \text{Normal}(\,0\,,\;0.5\,)
\end{aligned}
$$

The smooth basis function makes use of a penalised regression spline. The $\sigma_{\text{spline-coefficients}}$ term refers to the standard deviation of the spline coefficients, which controls the wiggliness of the spline. The $\alpha_{\text{spline-coefficients}}$ term refers to the fixed effects term, which describes the magnitude of the influence of the smooth term around the estimated mean value. For the half-Student-t distributions, the first

parameter represents the degrees of freedom, ν, followed by the location, μ and scale, σ, parameters.

$$\sigma_{\text{spline-coefficients}} \sim \text{Half-Student-t}(\ 3,\ 0,\ 2.5)$$
$$\alpha_{\text{spline-coefficients}} \sim \text{Half-Student-t}(\ 3,\ 0,\ 4.0)$$

1.2.2 Priors for the PuMBA SRTM model

For all priors, we use the same terminology used in Matheson and Ogden, 2023.

Global Intercepts

Note that all priors are defined over the natural logarithms of the parameters, where the mean is shown first and the standard deviation second.

$$\begin{aligned}\alpha_{\mathsf{R}_1} &\sim \mathrm{Normal}(0\ ,\ 0.25)\\ \alpha_{\mathsf{k}_2'} &\sim \mathrm{Normal}(-2.3\ ,\ 0.25)\\ \alpha_{\mathsf{BP}_{\mathsf{ND}}} &\sim \mathrm{Normal}(1.5\ ,\ 0.25)\end{aligned}$$

Individual deviations

Differences between individuals were defined by specifying the primary pharmacokinetic parameters in one variance-covariance matrix.

$$\begin{aligned}\begin{bmatrix}\tau_{\mathsf{R}_1}\\ \tau_{\mathsf{k}_2'}\\ \tau_{\mathsf{BP}_{\mathsf{ND}}}\end{bmatrix} &\sim \mathrm{MVNormal}\left(\begin{bmatrix}0\\0\\0\end{bmatrix}, \sum\nolimits_{\mathsf{Subject}}\right)\\ \Sigma_{\mathsf{Subject}} &= \begin{bmatrix}\sigma_{\mathsf{R}_1} & 0 & 0\\ 0 & \sigma_{\mathsf{k}_2'} & 0\\ 0 & 0 & \sigma_{\mathsf{BP}_{\mathsf{ND}}}\end{bmatrix}\mathbf{R}_{\mathsf{Subject}}\begin{bmatrix}\sigma_{\mathsf{R}_1} & 0 & 0\\ 0 & \sigma_{\mathsf{k}_2'} & 0\\ 0 & 0 & \sigma_{\mathsf{BP}_{\mathsf{ND}}}\end{bmatrix}\\ \sigma_{\mathsf{R}_1} &\sim \text{Half-Normal}(0\ ,\ 0.3)\\ \sigma_{\mathsf{k}_2'} &\sim \text{Half-Normal}(0\ ,\ 0.3)\\ \sigma_{\mathsf{BP}_{\mathsf{ND}}} &\sim \text{Half-Normal}(0\ ,\ 0.3)\\ \mathbf{R}_{\mathsf{Subject}} &\sim \mathrm{LKJ}(1)\end{aligned}$$

Regional deviations

For $\log BP_{\text{ND}}$ and $\log R_1$, regional differences were defined as unpooled effects using a dummy (indicator) variable defined with reference to the dorsolateral prefrontal cortex as covariates. For simplicity, all regional differences were defined for all other regions as zero-centred regularising priors with the same SD.

$$\beta_{R_1} \sim \text{Normal}(0\ ,\ 0.3)$$
$$\beta_{BP_{\text{ND}}} \sim \text{Normal}(0\ ,\ 0.3)$$

For k_2', regional differences were defined as a partially-pooled variable, arising from a common distribution.

$$\sigma_{\text{k}_2'} \sim \text{Half-Normal}(0\ ,\ 0.1)$$

Covariates for PK Parameters

For the effects of group on BP_{ND}, we defined priors for each region and diagnosis separately in order to detect regionally-specific effects.

$$\beta_{\text{Group} \times \text{Region}, BP_{\text{ND}}} \sim \text{Normal}(\ 0\ ,\ 0.2\ )$$

We also defined priors for age (per decade, centred) and sex.

$$\beta_{\mathsf{Age,k_2'}} \sim \mathrm{Normal}(\,0\,,\,0.1\,)$$
$$\beta_{\mathsf{Age},BP_{\mathsf{ND}}} \sim \mathrm{Normal}(\,0\,,\,0.1\,)$$

$$\beta_{\mathsf{Sex,k_2'}} \sim \mathrm{Normal}(\,0\,,\,0.05\,)$$
$$\beta_{\mathsf{Sex},BP_{\mathsf{ND}}} \sim \mathrm{Normal}(\,0\,,\,0.05\,)$$

Residual deviations

We defined residuals, ϵ, also as being correlated across the PK parameters in the same way as the individual and regional deviations. The residual variance hence corresponds to a combination of the TAC deviations described above for the SiMBA models, as well as inaccuracy in the estimation of these parameters.

$$\begin{bmatrix} \epsilon_{\mathsf{R_1}} \\ \epsilon_{\mathsf{k_2'}} \\ \epsilon_{\mathsf{BP_{ND}}} \end{bmatrix} \sim \mathrm{MVNormal}\left(\begin{bmatrix} 0 \\ 0 \\ 0 \end{bmatrix}, \textstyle\sum_{\mathsf{Subject}} \right)$$
$$\Sigma_{\mathsf{Subject}} = \begin{bmatrix} \sigma_{\mathsf{R_1}} & 0 & 0 \\ 0 & \sigma_{\mathsf{k_2'}} & 0 \\ 0 & 0 & \sigma_{\mathsf{BP_{ND}}} \end{bmatrix} \mathbf{R}_{\mathsf{Subject}} \begin{bmatrix} \sigma_{\mathsf{R_1}} & 0 & 0 \\ 0 & \sigma_{\mathsf{k_2'}} & 0 \\ 0 & 0 & \sigma_{\mathsf{BP_{ND}}} \end{bmatrix}$$

$$\sigma_{\mathsf{R_1}} \sim \text{Half-Normal}(0\,,\,0.1)$$
$$\sigma_{\mathsf{k_2'}} \sim \text{Half-Normal}(0\,,\,0.1)$$
$$\sigma_{\mathsf{BP_{ND}}} \sim \text{Half-Normal}(0\,,\,0.1)$$
$$\mathbf{R}_{\mathsf{Subject}} \sim \mathrm{LKJ}(2)$$

1.3 Supplementary Materials S3: Summary of Group Differences

1.3.1 Fixed Regional Effects Results

Below are shown the regional differences as shown in Figure 2.

Table S1: Summary of Group Difference Results: Estimate $\left(P\left[\hat{\beta} > 0\right]\right)$

Comparison	Region	2TCM BP_{F}	2TCM BP_{P}	2TCM BP_{ND}	SRTM BP_{ND}
NRM - HV	ACC	3.9% (91.1%)	6.1% (97.1%)	6.1% (98.5%)	-0.8% (58.6%)
NRM - HV	Amygdala	3.2% (86.6%)	5.3% (95.3%)	5.2% (95.2%)	-2.7% (75.5%)
NRM - HV	DLPFC	3.8% (91.5%)	6.1% (97.6%)	6.1% (98.1%)	-0.9% (59.5%)
NRM - HV	Hippocampus	1% (64.5%)	3.2% (83.5%)	3.2% (84.8%)	-5% (89.7%)
NRM - HV	Insula	3% (85.4%)	5.1% (95.3%)	5.1% (96.1%)	-2.4% (73.4%)
NRM - HV	MPFC	4% (91.8%)	6% (97.1%)	5.9% (97.7%)	-0.7% (57.6%)
NRM - HV	PCC	4.3% (93.1%)	6.5% (97.7%)	6.6% (99%)	-0.7% (57.9%)
NRM - HV	Parahippocampus	3.3% (86.4%)	5.5% (96.5%)	5.4% (97%)	-2.6% (74.8%)
NRM - HV	RN	8.7% (99.6%)	10.9% (99.9%)	10.9% (>99.9%)	13.3% (>99.9%)
NRM - AE	ACC	3% (82.7%)	3.8% (87%)	4% (90.4%)	4.2% (83.5%)
NRM - AE	Amygdala	3.8% (87.3%)	4.6% (90.8%)	4.8% (93%)	3.9% (82%)
NRM - AE	DLPFC	2.3% (77.1%)	3.1% (82.8%)	3.3% (86.5%)	3.6% (79.3%)
NRM - AE	Hippocampus	4.7% (92%)	5.5% (93.8%)	5.9% (96.8%)	3.9% (82%)
NRM - AE	Insula	3.9% (87.5%)	4.7% (92.1%)	4.9% (94.1%)	4.5% (84.7%)
NRM - AE	MPFC	2.8% (81.2%)	3.5% (85.3%)	3.7% (88.5%)	4.2% (83.6%)
NRM - AE	PCC	3.4% (84.7%)	4.2% (89.2%)	4.5% (92.1%)	4.5% (84.7%)
NRM - AE	Parahippocampus	4.5% (91%)	5.3% (94.4%)	5.5% (95.5%)	4.4% (84.8%)
NRM - AE	RN	8.5% (99.2%)	9.2% (99.4%)	9.5% (99.7%)	10.2% (99.1%)
AE - HV	ACC	0.9% (62.6%)	2.2% (78.1%)	2% (74.8%)	-4.8% (89.7%)
AE - HV	Amygdala	-0.5% (55.8%)	0.7% (59%)	0.4% (53.8%)	-6.4% (95.4%)
AE - HV	DLPFC	1.5% (70.4%)	3% (86.1%)	2.7% (83%)	-4.4% (86.7%)
AE - HV	Hippocampus	-3.5% (88.4%)	-2.2% (78.4%)	-2.6% (81.6%)	-8.6% (99.1%)
AE - HV	Insula	-0.9% (60.5%)	0.3% (54.1%)	0.1% (51.7%)	-6.7% (95.8%)
AE - HV	MPFC	1.1% (66%)	2.4% (79.6%)	2.1% (78.7%)	-4.7% (89.2%)
AE - HV	PCC	0.8% (62.2%)	2.2% (77.6%)	2% (75.8%)	-5% (90.6%)
AE - HV	Parahippocampus	-1.1% (63.2%)	0.2% (51.1%)	-0.1% (51.8%)	-6.7% (96.1%)
AE - HV	RN	0.2% (54.1%)	1.5% (67.8%)	1.3% (66.9%)	2.8% (75.3%)

1.3.2 Random Regional Effects Results

Below are shown the regional differences from the additional models fit with random slopes for regional differences in projection regions.

Table S2: Summary of Group Difference Results: Estimate $\left(P\left[\hat{\beta} > 0\right]\right)$

Comparison	Region	2TCM BP_{F}	2TCM BP_{P}	2TCM BP_{ND}	SRTM BP_{ND}
NRM - Control	Raphe	9.6% (99.7%)	11.1% (>99.9%)	11.5% (>99.9%)	11.6% (99.4%)
NRM - AE	Raphe	8.7% (98.7%)	9.8% (99.5%)	9.9% (99.1%)	10.2% (98.3%)
AE - Control	Raphe	0.8% (59.1%)	1.2% (63.6%)	1.5% (66.2%)	1.3% (60.9%)
NRM - Control	Projection	4.2% (91.4%)	5.8% (97.7%)	6.2% (98.5%)	-3.4% (18.7%)
NRM - AE	Projection	3.9% (90.2%)	4.9% (95%)	5% (95.5%)	4.2% (85.7%)
AE - Control	Projection	0.4% (54.5%)	0.9% (62.1%)	1.1% (65%)	-7.3% (2.6%)

And below are the comparisons between the raphe and projection region differences between groups, i.e. comparing whether the group differences were statistically differentiably larger in the raphe nuclei compared to the average group differences in projection regions. Here the differences are presented in the units of logBP.

Table S3: Summary of Regional Differences in Group Difference Estimates

Outcome	Group Comparison	Region Comparison	Difference $\left(P\left[\hat{\beta} > 0\right]\right)$
2TCM BP_{F}	NRM - Control	Raphe - Projection	0.050 (99.5%)
2TCM BP_{P}	NRM - Control	Raphe - Projection	0.049 (98.9%)
2TCM BP_{ND}	NRM - Control	Raphe - Projection	0.049 (99.3%)
SRTM BP_{ND}	NRM - Control	Raphe - Projection	0.050 (99.5%)
2TCM BP_{F}	AE - Control	Raphe - Projection	0.004 (57.2%)
2TCM BP_{P}	AE - Control	Raphe - Projection	0.003 (55.8%)
2TCM BP_{ND}	AE - Control	Raphe - Projection	0.003 (54.4%)
SRTM BP_{ND}	AE - Control	Raphe - Projection	0.004 (57.2%)

1.4 Supplementary Materials S4: Binding Potential Values

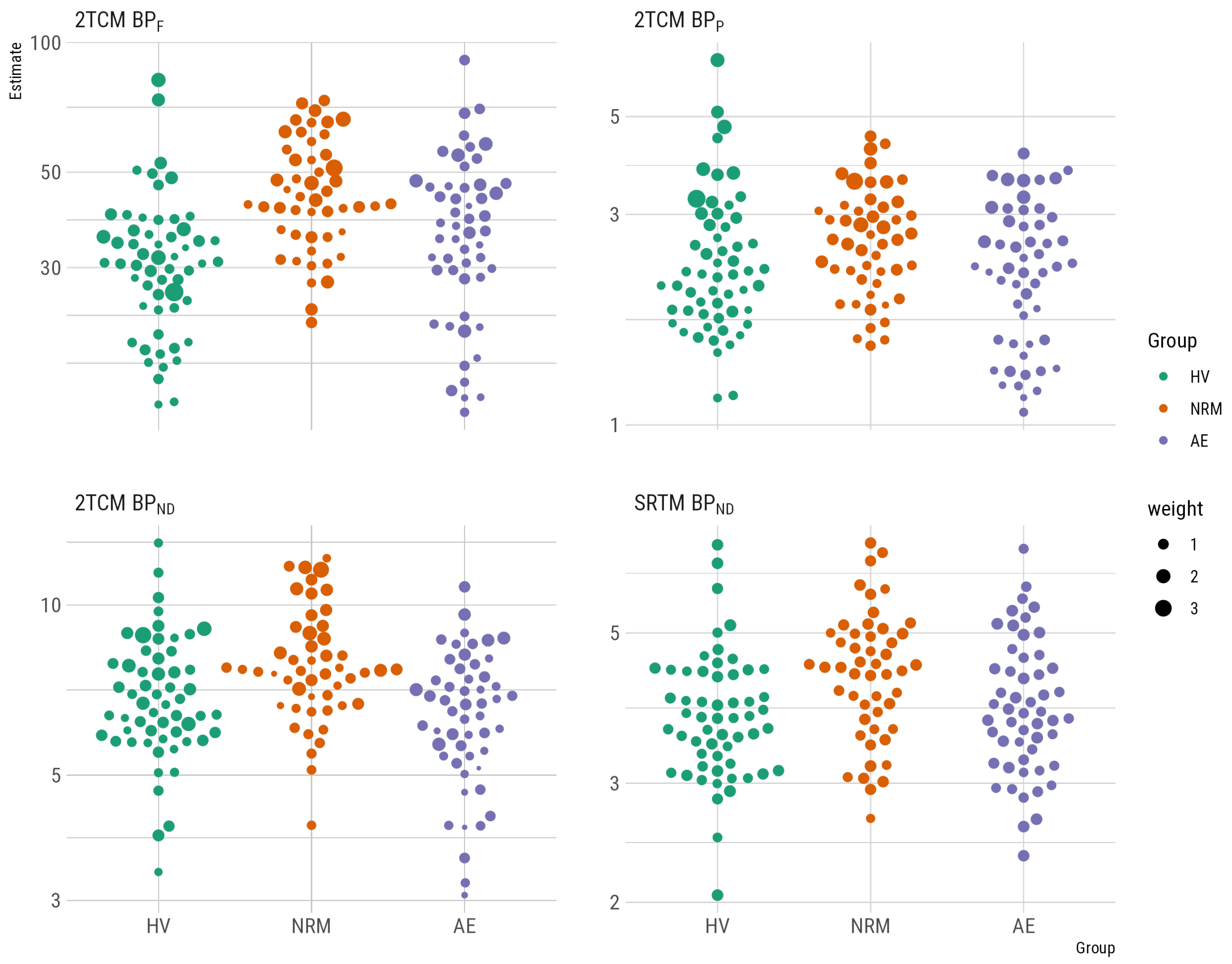

Figure S1: Raphe nuclei binding potential values for all outcome measures

1.5 Supplementary Materials S5: Raphe Nuclei Binding Potential Correlations

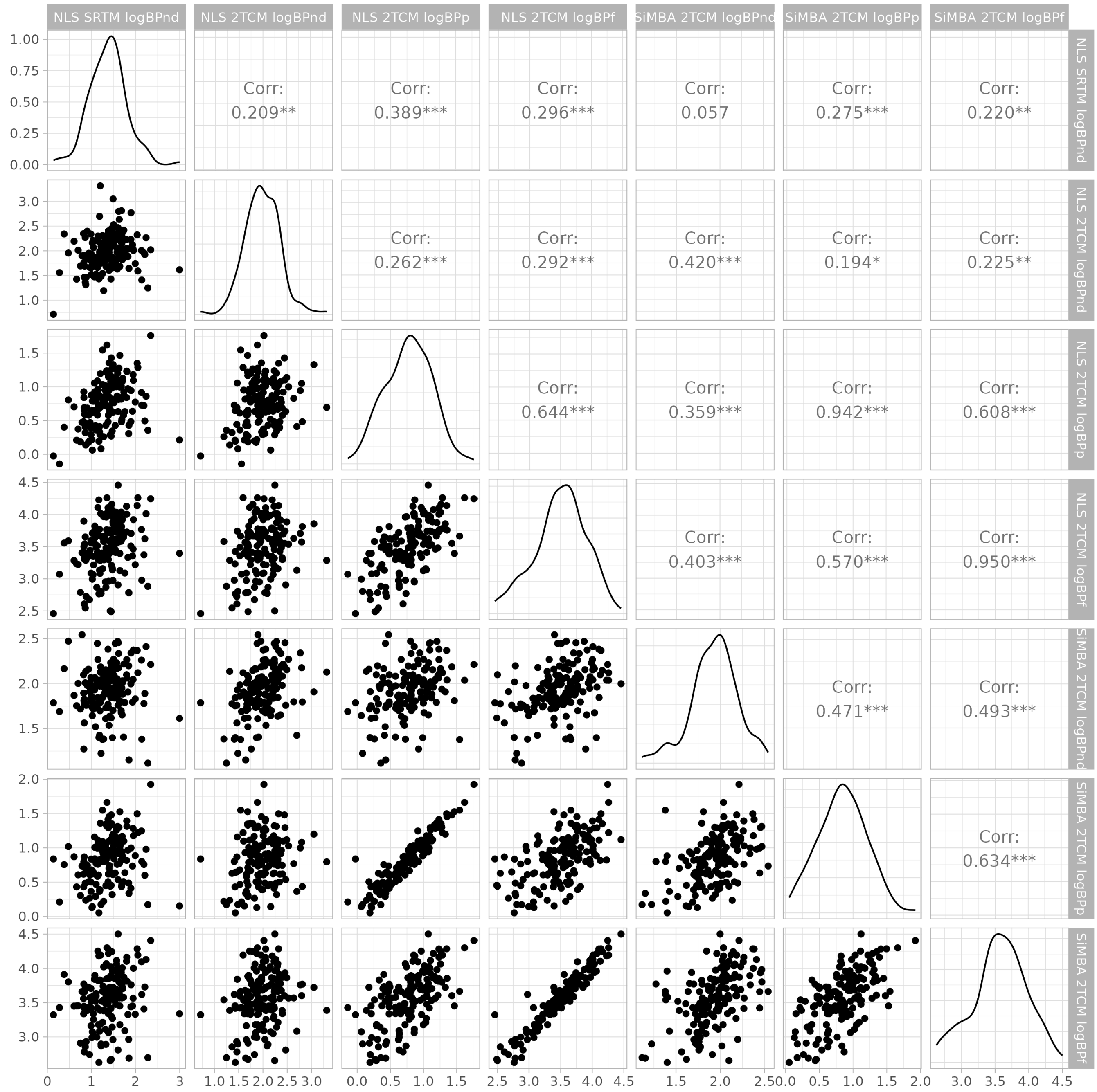

Figure S2: Raphe nuclei binding potential correlations for all outcome measures

1.6 Supplementary Materials S6: Correlations between Outcome Measures for each Method

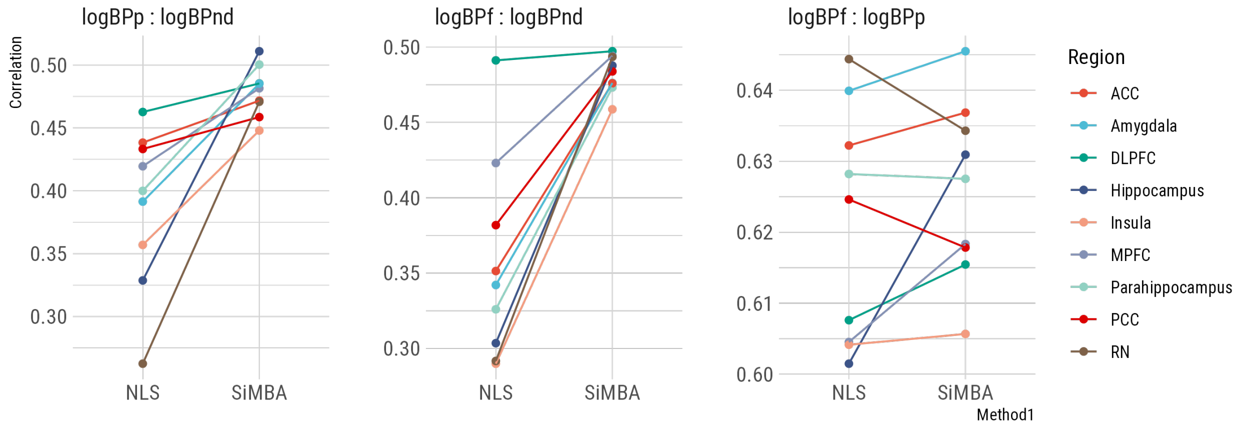

Figure S3: Comparison of outcome correlations for each method
